# Quantifying compressive forces between living cell layers and within tissues using elastic round microgels

**DOI:** 10.1038/s41467-018-04245-1

**Published:** 2018-05-14

**Authors:** Erfan Mohagheghian, Junyu Luo, Junjian Chen, Gaurav Chaudhary, Junwei Chen, Jian Sun, Randy H. Ewoldt, Ning Wang

**Affiliations:** 1 Laboratory for Cellular Biomechanics and Regenerative Medicine, Department of Biomedical Engineering, College of Life Science and Technology, Huazhong University of Science and Technology, Wuhan, Hubei 430074 China; 2 Department of Mechanical Science and Engineering, University of Illinois at Urbana-Champaign, Urbana, IL 61801 USA

## Abstract

Increasing evidence shows that mechanical stresses are critical in regulating cell functions, fate, and diseases. However, no methods exist that can quantify isotropic compressive stresses. Here we describe fluorescent nanoparticle-labeled, monodisperse elastic microspheres made of Arg-Gly-Asp-conjugated alginate hydrogels (elastic round microgels, ERMGs). We generate 3D displacements and calculate strains and tractions exerted on an ERMG. Average compressive tractions on an ERMG are 570 Pa within cell layers and 360 Pa in tumor-repopulating cell (TRC) colonies grown in 400-Pa matrices. 3D compressive tractions on a 1.4-kPa ERMG are applied by surrounding cells via endogenous actomyosin forces but not via mature focal adhesions. Compressive stresses are substantially heterogeneous on ERMGs within a uniform cell colony and do not increase with TRC colony sizes. Early-stage zebrafish embryos generate spatial and temporal differences in local normal and shear stresses. This ERMG method could be useful for quantifying stresses in vitro and in vivo.

## Abstract (web summary)

Increasing importance is placed upon the effect of mechanical forces on cell regulation, fate and disease states. Here, the authors describe a deformable fluorescent nanoparticle labeled elastic microsphere which can be used to calculate strain and traction forces in vitro and in vivo.

## Introduction

Increasing evidence suggests that mechanical forces are critical in regulating processes in development, physiology, and diseases^1–5^. For example, anisotropic forces in Drosophila embryos drive tissue elongation^6^; forces play important roles in stem cell differentiation^7,8^ and in organized germ layer patterning in mammalian embryogenesis^9^. Mechanical forces also appear to be important in cancer progression^10–13^. However, quantifying mechanical forces generated by living cells is not trivial. Forces have been inferred based on changes in cell shapes^14–16^. Several other methods can estimate tractions (interfacial stresses) in living cells or constituted tissues on 2D surfaces^17–21^ and in 3D matrices^13,22,23^ or estimate intercellular pressure^24^, but only the oil droplet method can quantify local traction variations in living embryonic tissues in situ^25^. In this method, anisotropic normal stresses of living tissues exerted on the oil droplet deform the droplet and changes its shape. By knowing the mechanical properties of the droplet membrane, one can estimate the stresses generated by the tissues. However, since the oil droplet is filled with incompressible liquid, this method cannot measure shear and isotropic compressive or tensile stresses generated in living tissues or between living cells. Besides tensile and shear stresses, compressive stresses have been shown to activate genes like TWIST in embryogenesis^26^ and to initiate cancer^27^.

To fill the gap of quantifying isotropic compressive, tensile stresses, and shear stresses, we present a strategy to develop an elastic round microgel (ERMG) method that can quantify normal and shear tractions between living cells layers and within living tissues. We find that compressive stresses are generated by actomyosin-dependent forces of the surrounding cells and are heterogeneous on ERMGs within a uniform cell colony and in an early-stage zebrafish embryo.

## Results

### Fabrication of ERMGs

**Fig. 1:**
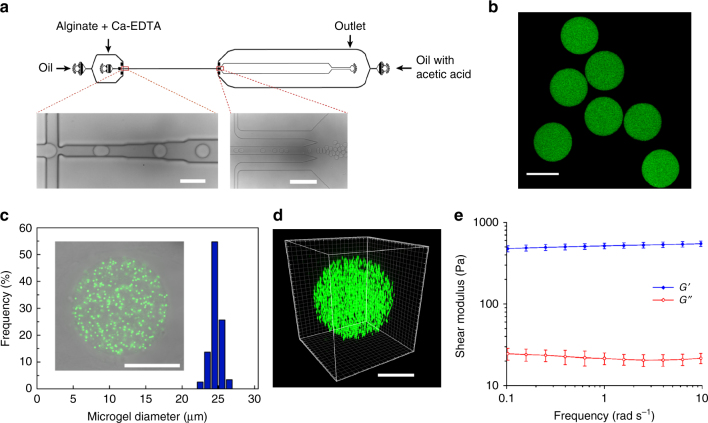
Fabrication of alginate microgels. **a** Schematic and microscopic images of the microfluidic device for alginate droplet generation. Right and left scale bares are 200 and 50 µm, respectively. **b** All alginate microgel droplets are uniform in size. Scale bar, 25 μm. **c** Confocal section and size distribution of the homogenous, 200-nm fluorescent nanoparticles-labeled elastic round microgel (ERMG). **d** 3D confocal image of an ERMG. Scale bars in **c** and **d**, 15 µm. **e** Characterization of rheological properties of alginate hydrogels using shear rheology. Mean ± s.e.m.; *n* = 6 hydrogel samples

Here, we fabricated biocompatible, monodisperse, homogenous, and elastic round alginate microgels with the size range of 15–30 μm via a droplet-based microfluidic device (Fig. 1a). The sodium alginate was first conjugated with RGD (Arg-Gly-Asp) to provide the integrin-binding sites for cell adhesion. We emulsified RGD-coupled alginate solution in oil phase at flow focusing junction of a microfluidic device and ionically cross-linked alginate with calcium ion to form microgels. To achieve a highly homogenous structure during gelation, a uniform mixture of alginate and calcium–EDTA (ethylenediaminetetraacetic acid) complex was introduced into the device. At pH of 7.4, Ca^2+^ was trapped between EDTA making it inaccessible to cross-link alginate polymers. Then gelation was initiated further downstream of the device by merging a new channel containing acidic oil (mixture of acetic acid and perfluorinated carbon oil) to the main stream. The acidic environment initiated the gelation process by releasing Ca^2+^ from the calcium–EDTA complex. This pre-gelation approach within the device resulted in better monodispersion and easier collection of the droplets than the external gelation approach that could lead to droplet coalescence before gelation. In addition, we found that this controllable internal gelation method was better in generating an internal uniform structure than an external gelation method using calcium chloride or an internal gelation method with calcium carbonate nanoparticles^28^. In a confocal image, FITC (fluorescein isothiocyanate) isomer I-labeled alginate round microgels that did not contain the fluorescent nanoparticles appeared to be homogeneous (Fig. 1b; Supplementary Fig. 1) and monodisperse (Fig. 1c); both are essential features for calculations of tractions exerted onto these round microgels. Transmission electron microscopy (TEM) images of fluorescent nanoparticles embedded microgels confirmed the validity of homogenous isotropic assumption for the microgels (Supplementary Fig. 2).

Fluorescent nanoparticles of ~200-nm in diameter were embedded in the microgels as a marker to track cell-induced deformation using fast iterative digital volume correlation (FIDVC)^29^. The nanoparticles were mixed in the alginate-calcium-EDTA solution at an optimum concentration without forming aggregation (Supplementary Fig. 1b). Reconstructed 3D confocal image showed uniform distribution of the fluorescent nanoparticles within the microgel (Fig. 1d). The alginate used in this study guaranteed the dominant elastic behavior over the viscous behavior, as the storage modulus was 15 times more than the loss modulus (Fig. 1e), consistent with published reports^30,31^. In addition to the nearly pure elastic behavior, frequency and strain independency of the gel allowed us to assume linear elasticity theory with constant elastic modulus in traction calculations from strains less than 30% (Fig. 1e; Supplementary Fig. 3a, b). The storage shear modulus of the hydrogel did not vary with the acetic acid concentration (Supplementary Fig. 3c) and was tuned from 0.5 to 1.2 kPa by varying the alginate and calcium concentration (Supplementary Fig. 3d). In addition, the aggregation and volume fraction of nanoparticles showed no significant effects on the storage modulus (Supplementary Fig. 4). Poisson ratio was determined via stretching alginate gels and found to be 0.4 (Supplementary Fig. 5). Thus Young’s moduli of the alginate hydrogels could be tuned between 1.4 and 3.36 kPa.

### Traction calculation and characterization

We determined full-field 3D deformation of the ERMG with respect to its stress-free state after cell lysis using Triton X-100 or after actin cytoskeleton disruption using Latrunculin A. The complete three-dimensional strain and stress tensor were calculated using the displacement gradient tensor and assuming a linear isotropic elastic material for the microgel. Using the stress tensor field and the reconstructed surface mesh of the ERMG, surface tractions were determined. To validate our computation method, we analyzed resolution and sensitivity using experimentally relevant simulated traction fields (Supplementary Fig. 6). Periodic compressive tractions with different wavelengths were applied on the surface of an elastic microsphere, and the tractions were recovered (Supplementary Figs. 7 and 8). The recovered displacements matched the simulated ones, and the results showed that the method can reliably measure 200 Pa traction at 18.85 µm length scale with traction error of less than 25% using microgels of 1600-Pa Young’s modulus (Supplementary Fig. 9a, b)). For each of the simulated loadings, the effect of noise was evaluated by superimposing the Gaussian white noise to the simulated displacement field with different signal to noise ratios. We measured the experimental noise by imaging the isolated ERMG in the fibrin gel using the same experimental setup, and found that displacement noises are 2–3 orders of magnitudes less than the displacements generated by living cells. (Supplementary Fig. 10). The traction recovery showed minor sensitivity to the noises in the range of experimental noise level (Supplementary Fig. 11a–d). In addition, the effect of fluorescent nanoparticle density on the recovered tractions was evaluated for bead densities ranging from 0.001 to 0.006 per voxel (including the actual experimental nanoparticle density of 0.003). The traction recovery exhibited little sensitivity with respect to nanoparticle density (Supplementary Fig. 9c) and sizes of microgel surface meshes (Supplementary Fig. 12).

### Traction quantification with ERMGs in multilayered cells

**Fig. 2:**
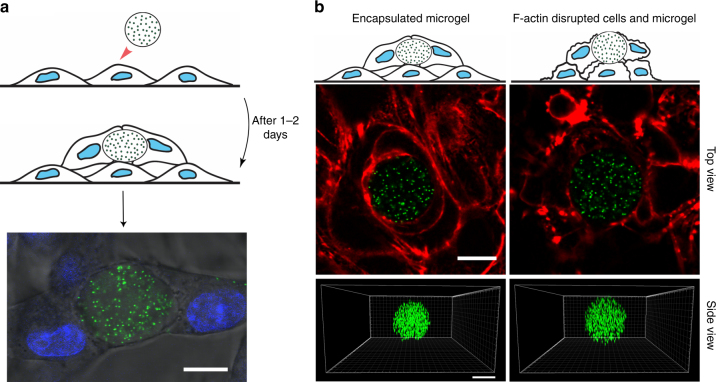
Elastic round microgels in multilayered melanoma cells. **a** Top and middle panels, experimental procedures. Bottom panel, a microgel (Young’s modulus of 1440 Pa) encapsulated by cells. Note that the microgel is deformed. Blue, nuclei of the cells. Scale bar, 25 µm. **b** Top and side views of 3D confocal images of the same microgel between untreated live cells (left panel) and Latrunculin A (1 µM for 90 min) treated cells (right panel). The microgel is compressed in the untreated condition. Green, 200-nm fluorescent nanoparticles embedded inside the ERMG. Red, filamentous actin staining. Scale bars, 15 µm

**Fig. 3:**
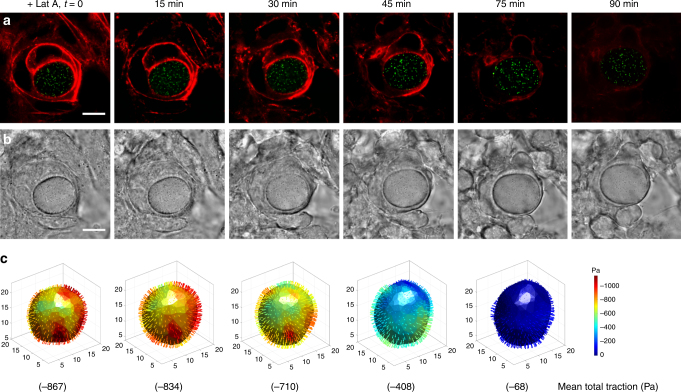
Tension in F-actin rings around the microgel is responsible for compression. **a** Representative fluorescent images show that treatment with Latrunculin A (1 μM final concentration) gradually dissipated F-actins (red) around the microgel, followed by the expansion of the microgel (green). **b** The corresponding bright field images of the embedded microgel (Young’s modulus of 1440 Pa) in multilayered cells. **c** 3D total traction maps and its mean value calculated with respect to its reference time at 90 min. The 3D traction maps show that the microgel was gradually decompressed as the filamentous actins disappeared. All scale bars, 10 µm

**Fig. 4:**
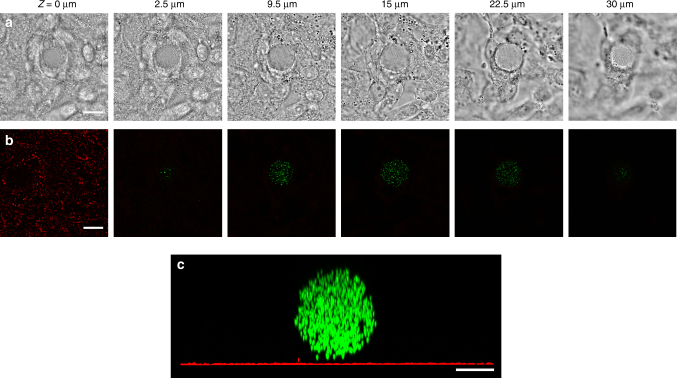
No mature focal adhesions are observed near a microgel in multilayered cells. **a** Representative brightfield confocal sections of the embedded microgel in multilayered cells. **b** Corresponding images of paxillin (red) and of the microgel (green). Focal adhesions are visible only at the bottom of the cell layer on top of the glass. Scale bars, 15 µm. **c** *Y–Z* projection of the 3D confocal fluorescent images of the microgel (green) and paxillin (red) shows that no mature focal adhesions or focal complexes are around the microgel. Scale bar, 10 µm

**Fig. 5:**
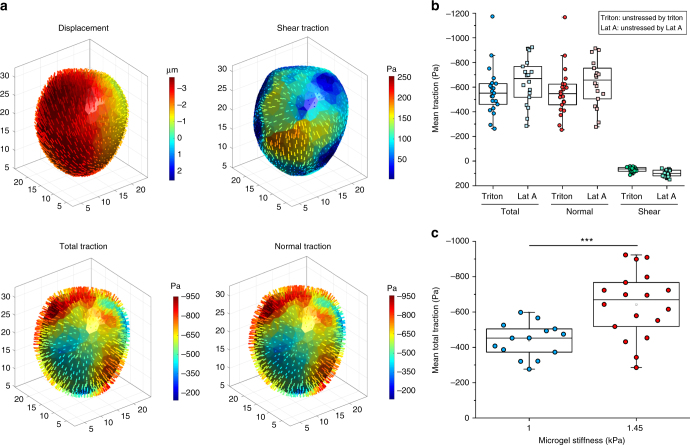
Quantification of tractions generated by multilayered cells. **a** An example of deformation and tractions on the elastic round microgel (ERMG) exerted by the multilayered cells. **b** Summarized data of total, normal, and shear tractions applied onto the ERMGs (Young’s modulus of 1.45 kPa). *n* = 21 ERMGs for Triton-treated cells and *n* = 18 ERMGs for Lat A-treated cells. **c** Scatter plot of mean total traction with respect to Young’s modulus of the microgels. The tractions were calculated using 1 µM Latrunculin A-treated samples as the unstressed reference condition. Solid lines in the box plots correspond to 25th percentile, median, and 75th percentile; *n* = 14 for 1 kPa; *n* = 18 for 1.45 kPa. ***P < 0.001. One-tailed Student’s *t*-test was used

We next used the ERMG method to measure cellular tractions in multilayered cells. Microgels were added onto the confluent layer of melanoma B16-F1 cells. After 1–2 days, cells formed a second layer encapsulating the microgel (Fig. 2a). Microgels without RGD modification showed no cell attachment and no encapsulation (Supplementary Fig. 13). 3D confocal stacks of an encapsulated microgel showed its cell-induced deformation and compressed state (Fig. 2b, left). Actomyosin-mediated cellular forces appear to be responsible for the compression of the ERMG, since disruption of F-actin with Latrunculin A returned the ERMG back to its round uncompressed state (Fig. 2b, right) and caused time-dependent reduction in compressive tractions in the ERMG from −867 to −68 Pa in 90 min (Fig. 3) (a minus sign here represents compressive stress). To investigate whether the cellular forces were applied via focal adhesions, we stained focal adhesion protein paxillin in these cells that might surround the ERMG. Interestingly, paxillin staining was only visible at the base of the bottom layer of the multilayered cells and no focal adhesions were visible in the cells near the ERMG (Fig. 4). These results suggest that the compressive tractions generated by living cells around the microgels in the 3D cultures are not applied via classical mature focal adhesions, dramatically different from the 2D cultures where focal adhesion are responsible for local high tractions^32^. We mapped displacements (deformation), shear, normal, and total tractions over the microgel surface (Fig. 5a). The averages of total and normal tractions over the microgel surface were −567 and −561 Pa when the cells were permeabilized with Triton, and −642 and −631 Pa when the endogenous forces were inhibited with Latrunculin A to disrupt F-actin to obtain the zero stress reference point (Fig. 5b). The slightly lower traction values associated with Triton treatment than with Latrunculin A might be due to the residual passive tension in the actin cytoskeleton. These traction values are in the same order of magnitude as the endogenous growth stress of tumors at early stages^27^. The average shear traction was only ~68 Pa for Triton-treated cells and ~100 Pa for Latrunculin A-treated cells, suggesting that normal traction is the dominant mode of applied tractions on the ERMG. Lowering the microgel modulus from 1.45 to 1 kPa decreased the total traction from −642 to −440 Pa (Fig. 5c), suggesting that when the cells came into contact with soft microgels the cells generated lower compressive stresses around them. For all measurements, static equilibrium of forces was evaluated by summing the surface forces for the microgel divided by the total force as sum of the absolute value of the surface forces:

1 $${\mathbf{error}}_{x,y,z} = \frac{{\left| {{\sum} {{\mathbf{F}}_{x,y,z\;{\mathrm{for}}\;{\mathrm{all}}\;{\mathrm{surfaces}}}} } \right|}}{{{\sum} {\left| {{\mathbf{F}}_{x,y,z\;{\mathrm{for}}\;{\mathrm{all}}\;{\mathrm{surfaces}}}} \right|} }}{\mathrm{X}}100$$

The result showed the average errors of 6.92%, 6.33%, and 16.45 % of the total force in *x*, *y*, and *z* directions, respectively.

### Cell traction in a tumor spheroid

**Fig. 6:**
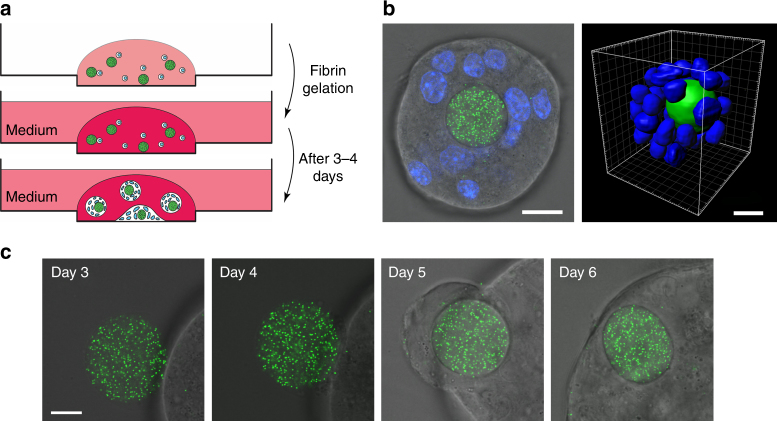
Elastic round microgels embedded in a melanoma cell colony. **a** Schematics of steps in preparing an elastic round microgel (ERMG) encapsulated in a melanoma colony of tumor-repopulating cells (TRCs) (spheroid) in 3D fibrin gels. **b** A confocal section of an embedded microgel (Young’s moduli of 1440 Pa) and its corresponding three dimensional rendering view (green, microgel; blue, nucleus). **c** Encapsulation processes of an ERMG by melanoma cells as a function of culturing time. Note shape changes of the ERMG while the cells are encapsulating it. All scale bars, 15 µm

We next used our method to measure cell-generated stresses within a 3D colony of melanoma tumor-repopulating cells (TRCs)^11^. We mixed ERMGs of ~25 µm in diameter with the cells and fibrinogen and plated the mixture on the glass-bottomed dishes. After forming the fibrin gel at 4 mg ml^−1^ fibrinogen with 100 U/ml thrombin (corresponding to 420 Pa in Young’s modulus^33^) and culturing the cells in the medium for 3–4 days, the cells next to an ERMG formed a round colony around the ERMG (Fig. 6a). Three-dimensional rendering view of the microgel from confocal sections confirmed the encapsulation of the ERMG by the cells (Fig. 6b). To visualize the encapsulation processes of the ERMG by the tumor cells in the colony, we imaged the selected colony in succeeding days (Fig. 6c). The encapsulation was initiated by forming a ring-like structure around the microgel, when the cells on each side of the microgel tried to attach to each other across the microgel. This encapsulation of the microgel inside the colony was associated with a compressive deformation (Fig. 6c). To quantify the compressive state of the encapsulated microgel, the colonies were treated with Triton to induce cell lysis to obtain stress-free condition (Supplementary Fig. 14). For the microgels in the fibrin gel but outside of the cell colonies, after Triton treatment, the tractions were ~15 Pa and there was no change in the microgel volume (Supplementary Fig. 15), much smaller than cell-generated tractions (200–600 Pa), suggesting Triton had little effect on the ERMGs themselves.

**Fig. 7:**
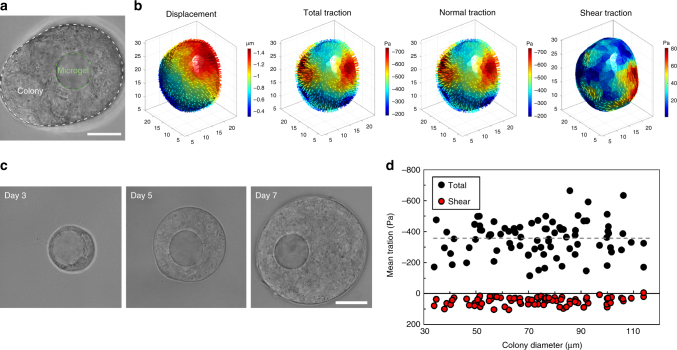
Tractions on the microgels do not increase with tumor colony sizes. **a** An example of an encapsulated ERMG (Young’s moduli of 1440 Pa) inside a 3D melanoma colony of TRCs (Day 6 of culture). The colony grew from a single melanoma cell at Day 0. Green dashed line, microgel; white dashed line, edges of the colony. **b** 3D deformation and corresponding tractions of the ERMG. **c** Representative images of three different melanoma colonies at different days of culture, each colony encapsulating an ERMG. Each colony (of all colonies) grew from a single melanoma cell at Day 0. **d** Average absolute total and shear traction on the surface of each ERMG as a function of colony size. Solid line, arithmetic mean of total tractions. Average total and shear tractions are −357.9 ± 12.03 Pa and 49.1 ± 2.58 Pa, respectively (mean ± s.e.m., *n* = 87 ERMGs). All scale bars, 25 µm

**Fig. 8:**
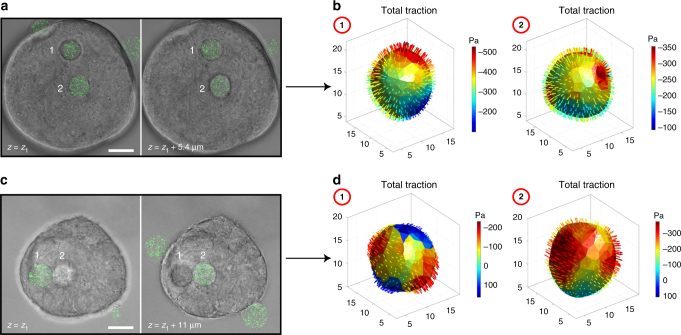
Spatial distribution of tractions on two microgels inside a melanoma cell colony. **a** Confocal sections of two embedded microgels (Young’s moduli of 1440 Pa) in different focal planes inside a round colony. **b** Corresponding 3D total traction maps of the two microgels. Note that peak tractions and traction distributions are different for these two microgels in this round colony. **c** Images of two embedded microgels (Young’s moduli of 1440 Pa) at two different *z* positions in another round colony. **d** Corresponding 3D total traction maps of the two microgels. Note that tensile tractions are apparent on parts of the microgel #1 and traction distributions are dramatically different for these two microgels in this round colony. All scale bars, 20 µm

Surprisingly, when the microgel was trapped inside a near-spherical melanoma colony, the traction distribution was non-uniform on the ERMG: local maximum compressive stresses were >3-fold of the local minimum compressive stresses (Fig. 7a, b). Published reports show that growth-induced solid stress in tumors increases with tumor sizes for tumor larger than 3 mm^12,34,35^. Colonies increased their diameters from 35 to 113 µm when the tumor cells were cultured from a few days to 6 days (Fig. 7c, d). No correlation was observed between the mean compressive traction and the colony size. The average total and shear tractions were −358 and 49 Pa, respectively, suggesting that compressive stresses dominate shear stresses inside the tumor cell colony. To examine traction variation in the same colony, we trapped two microgels in the same colony. We found that even though the colony appeared to be round and uniform, the local tractions generated by the cells in the colony were substantially different (Fig. 8). In addition, more than two microgels could be encapsulated and their tractions could be quantified within a single cell colony (Supplementary Fig. 16). Similar to what was observed with the ERMGs trapped in multilayered cells, no focal adhesions were found around the ERMGs trapped in 3D melanoma colonies (Supplementary Fig. 17), suggesting that the compressive tractions were not applied via classical mature focal adhesions under these conditions, fundamentally different from tractions generated by cells cultured in 2D conditions.

### Quantifying tractions in in vivo tissues

**Fig. 9:**
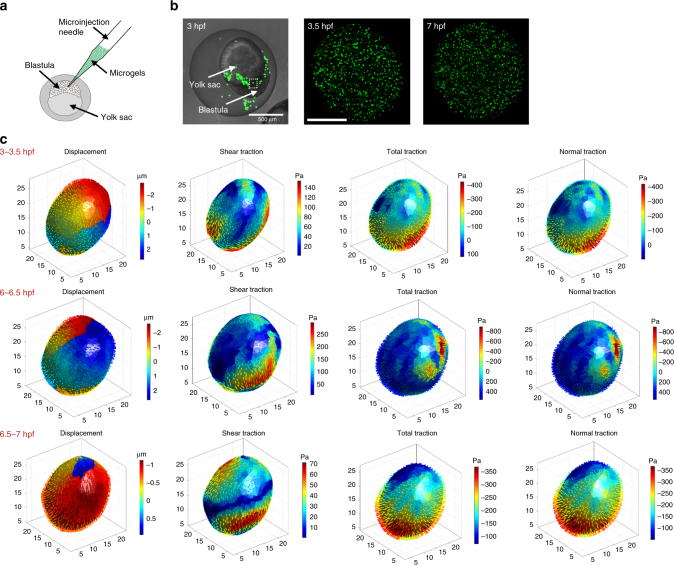
Quantifying tractions in a zebrafish embryo. **a** Schematic of microinjection of ERMGs onto a zebrafish embryo at the blastula stage by puncturing the outer membrane of the zygote and injecting into spaces surrounding the embryo. **b** A low-resolution confocal image (left) showing the whole embryo at the blastula stage (3 hpf, hours post-fertilization) and the injected ERMGs onto the embryo. The dashed white line box circles the microgel that is zoomed in for high-resolution imaging at different developmental stages: 3.5 (middle) to 7 (right) hpf. Scale bars: left, 500 µm; middle, 10 µm. **c** 3D maps of displacement (left), shear traction (middle left), total traction (middle right), and normal traction (right) of an ERMG. The 3D confocal images of the ERMG were taken starting at 3 hpf, then at 3.5, 6, 6.5, and 7 hpf, respectively. The 3–3.5 hpf images (top row), 6–6.5 hpf images (middle row), and 6.5–7 hpf images (bottom row) are different in displacements or tractions analyzed between those 30-min intervals. Note that since the zero traction condition cannot be obtained in live embryos, only changes in tractions can be measured

**Fig. 10:**
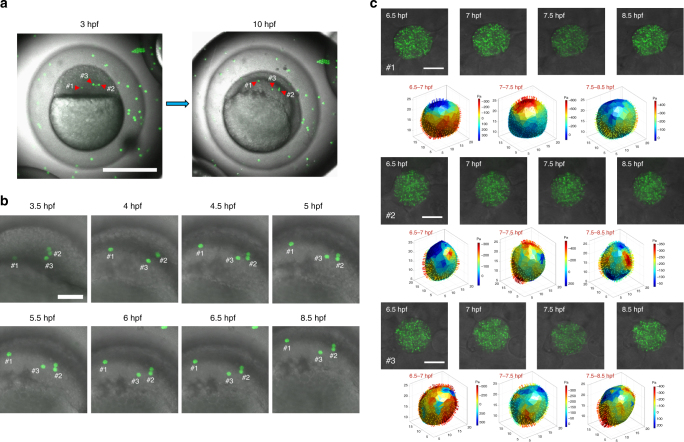
Substantial spatial and temporal traction variations inside a zebrafish. **a** A developing zebrafish embryo from 3 to 10 hpf with numerous embedded microgels, of which #1, #2, and #3 microgels are imaged and their tractions are calculated. Scale bar, 500 μm. **b** Corresponding high magnification confocal sections of the three embedded microgels at different stages of development at distinct locations. A microgel near #2 microgel is too blurry such that its tractions could not be quantified. Scale bar, 100 μm. **c** 3D plots of relative total tractions (changes in total tractions) for the corresponding microgels from 6.5 to 7, 7 to 7.5, and 7.5 to 8.5 hpf. For clarity, only total tractions are shown here. All scale bars, 10 μm

To determine if ERMGs can be used to quantify compressive stresses in living tissues, we microinjected ERMGs into an early blastula of a zebrafish embryo (Fig. 9a). As the embryo continued to develop from 3 (hours post fertilization, hpf) to 7 hpf (Fig. 9b), the cells in the embryo generated substantial stresses, as evident by the heterogeneous shear, tensile, and compressive stresses on an ERMG embedded in the embryo (Fig. 9c). Tensile stresses dominated the total stresses (tractions) on the ERMG by the surrounding cells from 6.0 to 6.5 hpf (Fig. 9c, middle panel), but compressive stresses became more dominant from 6.5 to 7.0 hpf (Fig. 9c, bottom panel). To examine traction variations in the same embryo, we followed three ERMGs at different positions in the same embryo and quantified their tractions as the embryo was developing (Fig. 10a, b). Interestingly, at the same developmental stages (e.g., 6.5 hpf to 7 hpf), three different microgels, separated spatially by only ~50–150 μm, exhibited substantial differences in 3D tensile and compressive tractions (Fig. 10c). In addition, traction maps changed greatly from 6.5 to 8 hpf for a single microgel (Fig. 10c). These results suggest that local forces varied extensively spatially and temporally with embryonic development. These results suggest that the ERMG method can quantify dynamic changes in stresses in living tissues.

## Discussion

It is rather challenging to quantify compressive stresses within layers of living cells or in living tissues. As far as we know, few methods can achieve this goal. The oil droplet method^25^ can quantify anisotropic normal stresses in living tissues. The main limitation of that method is that the oil droplet contains incompressible fluids and thus cannot quantify isotropic normal stresses (either tensile or compressive). In addition, that method cannot measure shear stresses. In contrast, our ERMGs are made of elastic materials that can quantify both isotropic normal stresses (tensile and compressive) and shear stresses. A recent report^24^ uses microspheres that are made of elastic polyacrylamide to measure intercellular pressures. While polyacrylamide is nontoxic, acrylamide is toxic to living cells; there is a possibility that acrylamide molecules diffuse out of the polyacrylamide to damage the cells, especially when the soft polyacrylamide gels (as a result of a low concentration cross-linking molecule bisacrylamide) are made. As such, it is rather challenging to use the polyacrylamide method to make soft elastic microspheres to have a modulus of a few hundred Pa. In contrast, we make our microspheres from alginate that is known to be nontoxic^36^ and can be as soft as a few hundred Pa, suitable for early embryogenesis studies when cells and tissues are very soft. In addition, the polyacrylamide microbeads are fabricated using a water-oil emulsion approach. As such, the beads are not monodisperse but vary in sizes. This makes the quantification of changes in stresses in different locations in cell layers or tissues very difficult. In contrast, we used a modified microfluidic channel approach that can fabricate every microbead to the same diameter (i.e., to be monodisperse). Being monodisperse is a critical requirement for computing reliable normal stresses and shear stresses in living cell layers and in living tissues. Furthermore, the polyacrylamide microbeads can only measure pressure distribution within cell spheroids in response to osmotic pressure changes via measuring volumetric changes in microspheres *(*Δ*V/V*). Thus no quantitation of stress distribution on the microsphere is possible, since no local strains (normal and shear) are quantified and Poisson ratio is not measured. In sharp contrast, we have trapped nano-sized fluorescent particles inside the microgels and developed a rigorous 3D strain and stress computational method from 3D displacement maps, whose robustness is validated by simulations. In terms of the biological significance, the ERMG approach could quantify local variations in stresses (tensile/compressive and shear), whereas it is not possible for the polyacrylamide microbead method to quantify local normal stress heterogeneity on different sides of the microsphere, generated by various nearby cells nor it is possible to quantify shear stresses. Since both shear stresses and isotropic tensile/compressive stresses have been implicated in many biological systems (ranging from embryonic development, to lung tissue expansion and compression during respiration, to bone tissue remodeling, and to cancer initiation and progression), our unique ERMG approach represents a major advance in concept, methodology, and potential application in biology. Recently a method of measuring 2D solid stress has been reported via planar cutting the (tumor) tissue and estimating the compressive stresses of the tumor tissue at the cutting plane^12^. While this method is useful, the tissue of interest must be excised to obtain strains and stresses normal to the cutting plane, whereas stresses at other directions or inside the tissue cannot be estimated. Hence it is not possible to quantify time-dependent changes of stresses in the same living tissue. In contrast, our ERMG method uses the strategy of an elastic microsphere. Our method can be used to quantify dynamic changes in compressive or tensile (either isotropic or anisotropic) and shear stresses generated by living cells and tissues. Since quantification of stresses depends on the measured deformation of the microsphere, light penetration is limited through thick living cell layers or living tissues, thus limiting the utility of the ERMG method. Our method of using fluorescently labeled ERMGs to quantify tractions in living cell layers and living tissues is based on the notion that fluorescent nanoparticles inside the ERMGs displace because of deformation of the ERMG and no slippage occurs between the nanoparticles and the alginate polymers. The no slippage assumption is reasonable since the pore size of the alginate polymers is only ~5 nm^37^ and the nanoparticles have a diameter of ~200 nm. In addition, the nanoparticles appear to follow the resumption of the deformed ERMG without any delay in time after the cell-generated stresses on the ERMG is abolished with treatment of Triton. All these lines of evidence suggest that fluorescent nanoparticle-labeled ERMGs can quantify tractions among living cells and in living tissues. Another issue with the ERMG method is that alginate is ionically cross-linked by calcium, and thus may be potentially sensitive to changes in calcium concentration in the environment. We find that when the ERMGs and the polymerized alginate are kept in deionized water for up to 36 days, the shape of the ERMGs do not change, the fluorescent nanoparticles do not leak out of the ERMG, and the alginate modulus do not vary much (Supplementary Fig. 3d). In addition, we have kept ERMGs in calcium-containing medium when the ERMGs are stored for months. It is also known that calcium concentration in the blood and in the interstitial fluid is relatively constant at 1–2 mM, suggesting that the cross-linked alginate in the ERMGs may not change much for long durations of several months in in vivo conditions.

Alginate polymer chains cannot be degraded and cleaved due to lack of the enzyme alginase in mammals to proceed this reaction. However, biodegradation of ionically cross-linked alginate is associated with exchange of divalent ion cross-linking the gel with monovalent cations, such as sodium ions^38^. Here, we analyzed the degradation of ERMG by quantifying its volume loss before encapsulation and after cell lysis. The volume initially decreased due to the compressive stress during encapsulation, however, after the cell lysis when the microgel was unstressed, it returned to its initial volume. As a result, no volume loss or degradation of the microgel was observed after its contact with living cells (Supplementary Fig. 18). A recently published report demonstrates the generation of thin tunable microgels to encapsulate single living cells for niche modeling and drug delivery^39^. Our method can be taken as an inside-out version of that approach since our microgels are outside of the cells and are used to quantify forces generated between the living cells.

It is interesting that although tractions around the ERMGs in multilayered cells and in a 3D cell colony are generated via cellular endogenous actomyosin forces, these stresses are not applied via focal adhesions since no classical mature focal adhesions are observed around the ERMG surfaces. This finding represents a major departure from what is known for tractions in cells on a 2D rigid surface, where focal adhesions are the major route of traction application^32^ and appears to mirror a result that no mature focal adhesions are found in cells embedded in a 3D soft matrix of type I collagen^40^. However, a follow-up study by a different group using a similar type I collagen concentration has observed classical focal adhesions in the 3D matrix^41^. Although discrepancies on the presence or the absence of mature focal adhesions in single cells in 3D between the two published studies remain to be resolved, our current observation is from multilayered cells or from a 3D cell colony where neighboring cells form cell–cell adhesions. To find out the underlying mechanisms of our observation, we performed additional experiments to determine whether it is surface chemistry, rigidity, or dimensionality that determines adhesion behaviors. We find that cells cultured on a thin alginate-RGD layer-coated 2D rigid surface developed mature focal adhesions, similar to those on fibronectin-coated 2D rigid surface. In contrast, cells on top of a thick alginate-RGD layer that has the same modulus as the ERMGs did not form mature focal adhesions but generated dotted and round focal plaques/complexes-like structures (Supplementary Fig. 19). Together with our observation that no mature focal adhesions and no round focal complexes are observed surrounding the ERMGs in a 3D culture, these results suggest that both ERMG modulus and dimensionality (2D vs. 3D) determine whether surrounding cells generate mature focal adhesions and focal complexes or not. However, it is possible that focal adhesion proteins themselves may still be involved in force transmission from the cells to the ERMGs. Moreover, since only RGD-conjugated alginate ERMGs can be trapped or encapsulated by the cells in a 3D culture, integrin-ECM interaction is still necessary for tractions exerted onto the ERMGs. While F-actin rings surrounding ERMGs appear to be responsible for generation of compressive tractions, future studies are needed to explore in detail the roles of integrin-mediated adhesion and other adhesion molecules in generating compressive and shear tractions in 3D. For example, compressive stresses on ERMGs may be generated by contracting actin cables (see Fig. 4) that link neighboring cells (i.e., cell–cell generated compressive forces are transferred to the ERGM) via cadherins^42^, whose specific roles need to be elucidated in the future.

How to quantify the absolute traction in living systems is not a trivial issue. Triton or Latrunculin A treatment to get unstressed shape of the ERMG is not feasible in vivo. This limitation can be mitigated by starting with the unstressed shape of the ERMG at time zero, when the microgel has not been trapped by living cells in vivo. This way, all other images of the deformed/stressed ERMG can be referenced to this unstressed shape. In general, relative traction changes can be quantified as a function of developmental stages and time in a live embryo.

In the current study, we have found that stress distributions on various ERMGs are different in the same cell colony or in a zebrafish embryo. It is not clear whether this difference arises from spatial variations in the stress fields within the same cell colony or the embryo, or whether the difference is due to local variations in how cells packed around individual ERMGs. All the ERMGs have the same size (monodisperse), the same mechanical properties (modulus), and the same chemical properties (the conjugations between alginate and RGD appear to be evenly distributed^43^). Therefore it would be hard to imagine that cells would choose to pack around one ERMG differently than around another ERMG. Instead, it is conceivable that spatial variations in the stress fields within the cell colony and the zebrafish embryo account for this difference. These variations of stress fields might originate from cells at different spatial locations being at different phases of cell cycles and/or at different contractile states. This hypothesis can be tested in the future.

Our results of microgels in multi-layered cells or in a cell colony show a substantial reduction in microgel volume, suggesting that compressive stresses are the dominant stresses exerting onto these microgels. Since there are F-actin rings from the surrounding cells around the microgel (Fig. 3), it is difficult to imagine an unconfined compression behavior responsible for the tensile stress on a microgel encapsulated in multi-layered cells. Therefore, it is possible that the tensile stress originates from integrin-based cell-microgel adhesion, which needs to be investigated in detail in the future. Inside a cell colony, sometimes both tensile and compressive stresses are exerting on the microgel (Fig. 8d); since distributions of tensile tractions and compressive tractions are not symmetric on the microgel, these results suggest that the traction pattern is not due to a pure tensile behavior alone, nor due to an unconfined compression behavior alone. It has been established that anisotropic tensile forces drive drosophila tissue elongation developing embryos^6^. However, quantification of tensile forces in embryos is accomplished only recently with the oil droplet method^25^. In a zebrafish embryo, we find that some microgels experience simultaneously both tensile and compressive stresses (Figs 9c and 10c), suggesting that both types of stresses are at play during development, extending the previous published work that anisotropic tensile stresses are critical for development^6^. In the future, it will be interesting to follow for a long period (say, a few days) of time the changes in stresses in the developing zebrafish embryo, and to determine how the stresses might vary at various locations at different developmental stages to drive and regulate embryogenesis.

Here we have used a polymer concentration between dilute and concentrated regimes (Supplementary Fig. 3e, f) to achieve an elastic shear modulus of 0.5–1.2 kPa. Using higher concentration of alginate (>1 wt%) leads to very stiff microgels, resulting negligible and unmeasurable cell-induced deformation. In addition, increasing the concentration of alginate solution to 1 wt% can result in jetting and poly-dispersion of the droplets due to an increase in the viscosity of aqueous solution in the microfluidic device.

Although the stiffness of the ERMG was adjusted to be soft enough to capture the cell-induced deformation without exhibiting a non-linear behavior, the maximum principal strain in small surface regions of some ERMGs in multilayered cells condition can go up to 40%. Based on the Lissajous curves (Supplementary Fig. 3b), the hydrogel at this strain exhibits non-negligible dissipation and the ratio of storage to loss modulus decreases from 15 to 6. To estimate to what extent tractions are underestimated, we have employed an Neo-Hookean model to calculate stresses and found that that the average compressive traction is increased by ~20% (from −567 to −687 Pa) onto ERMGs by the multilayered tumor cells, compared to that using the linear elasticity model. These results suggest that elastic behaviors still dominates over dissipative behaviors. A wider range of microgel modulus could be used for future studies depending on the expected traction forces and the modulus of the tissue environment of interest.

When an ERMG is encapsulated inside a round tumor colony that seems uniform, the quantified local compressive stresses on the ERMG is non-uniform. One possible explanation of this finding is that tumor cells surrounding the microgel generate different stresses, likely due to the possibility that these tumor cells are in different differentiation state^11^. Alternatively, local fibrin polymers form different networks locally outside the microgels, making it possible that the elastic stresses are different on the surface of the ERMG. Nevertheless, the underlying mechanism for this local heterogeneity in compressive tractions needs to be determined in the future. A recent report using the planar-cut method of exercised tumors shows that solid stress increases with tumor size, and that normal tissues surrounding the tumor are a major contributor to intratumoral solid stress^12^. The same report finds, however, that tumors with diameters of less than 3 mm do not increase solid stress with tumor size^12^. Our current finding that the mean compressive stress does not change when the tumor colony size increases from 30 (0.03 mm) to 110 μm (0.11 mm) is consistent with this report^12^, but appears to differ from the results from normal mouse tooth mesenchymal cell aggregates where anisotropic stresses increase with aggregate sizes^44^. Compared with the 2D planar-cut method^12^, our ERMG method can dynamically quantify 3D stresses in living normal and tumor tissues when the tumor size is very small («1 mm), and thus may be useful in determining force impact on early stages of tumor progression.

It is known that secondary metastatic sites for many malignant solid tumors are limited to only a few organs: bone marrow, brain, liver, and lung, a phenomenon called metastatic tropism^45^. These organs are in general very soft: bone marrow has a stiffness of ~100 Pa^1^, brain stiffness is ~200–1000 Pa^1^, liver stiffness is ~400 Pa^46^, and lung stiffness is 100–1000 Pa^47^. Although the underlying mechanism for metastatic tropism is unclear at this time, we have hypothesized that the soft undifferentiated TRCs proliferate rapidly at these soft secondary sites. In the present study, we have chosen 420-Pa stiffness of the 3D fibrin gels to embed the tumor cells to encapsulate the ERMGs, making the matrix stiffness similar in magnitudes as that in the soft organs. It will be interesting to explore why the average compressive stress (−360 Pa) generated by the undifferentiated melanoma TRCs in the 3D colony is lower than the average compressive stress (−570 Pa) generated by the differentiated melanoma counterpart cells in 3D multilayers. In the future, it would also be interesting to quantify compressive tractions at those secondary sites of tumor to explore their impact on tumor growth at the metastatic sites in vivo.

## Methods

### Alginate preparation

High molecular weight sodium alginate was purchased from WillPowder. The alginate was dialyzed against deionized water for three days (molecular weight cutoff of 3.5 kDa) followed by stirring overnight with 0.5 g activated charcoal per 100 ml of 1w/v% alginate in deionized water. The solution was then sterilized through a 0.22-μm filter and lyophilized for further modification. RGD-modified alginate was prepared by conjugation of GGGGRGDSP (Peptide International) to the purified alginate polymer using carbodiimide chemistry^43^. Briefly, 1 g of the purified alginate was reconstituted in MES buffer (0.1 M MES, 0.3 M NaOH, pH 6.5) at the concentration of 1% w/v. EDC (*N* -(3-dimethylaminopropyl)-*N*’-ethylcarbodiimide hydrochloride, Sigma-Aldrich) was added to activate the carboxylic acids of the polymer chain at 1:20 molar ratio to the uronic acid monomers of the alginate. Subsequently, Sulfo-NHS (*N*-hydroxysulphosuccinimide, Thermo Fisher Scientific) was added to the reaction at 1:2 molar ratio to the EDC followed by the addition of 150 µM peptide resulting in the coupling of 2 RGD peptide per alginate polymer on average. The coupling efficiency using this procedure was previously characterized using 125I labeled RGD peptides^43^. After reaction for 20 h while stirring, the solution was purified against unreacted peptide by dialyzing against deionized water for three days, mixing with 0.5 g activated charcoal, sterilized filtered (0.22-μm pores), freeze-dried, and reconstituted in deionized water. 0.8%w/w alginate solution was prepared by dissolving the RGD-modified alginate in deionized water. The calcium-EDTA solution was prepared by mixing 40 mM solution of calcium chloride (Sigma-Aldrich) and EDTA (Sigma-Aldrich) (1:1 volume ratio) followed by pH adjustment to 7.2 using a 1 M NaOH solution. Subsequently, fluorescent nanoparticles (0.2-µm in diameter, non-functionalized yellow–green; Polysciences) were mixed with calcium-EDTA solution (45 µl/ml), and a final solution was achieved by adding the same volume of alginate solution and calcium–EDTA–nanoparticles complexes. The solution was gently vortexed for several minutes to form homogenous mixture. Initially, we found that a number of fluorescent nanoparticles were diffused outside the ERMGs. We determined that this was due to the problem of carboxylated nanoparticles (that we originally used) that could not bind tightly to the alginate polymers, especially at the edge of the microgel. We improved the method by using non-functionalized fluorescent nanoparticles instead of carboxylate-modified ones and no diffused out nanoparticles were observed for embedded microgels in a melanoma cell colony or in multilayered cells.

### Florescent labeling of alginate

Alginate was labeled with fluorescence using carbodiimide chemistry following a previously reported protocol^48^. Briefly, alginate solution with 90 mM carboxyl group was mixed with 9 mM EDC followed by the addition of 9 mM Sulfo-NHS. The solution was kept for 2 h at room temperature with stirring. FITC isomer I (Sigma-Aldrich) was added to the concentration of 0.036 mM and reacted for 20 h at room temperature under stirring. To remove unreacted fluorescence, the solution was then dialyzed against deionized water at 4 °C overnight followed by three shifts in 1 M NaCl (molecular weight cut off 12–14 kDa). Finally, it was dialyzed against deionized water until losing its yellow color. The solution was then freeze dried and reconstituted in deionized water.

### Fabrication of microfluidic devices

Microfluidic device was manufactured using soft lithography^49^. Master was fabricated by spin coating negative photoresist SU8-2015 (Microchem Corp.) onto a 4-inch single side polished silicon wafer (Pure Wafer, San Jose, CA) to a final resist thickness of 30 μm. A flood exposure (Flood Exposure Model 60, ABM Inc.) was used to expose the photoresist by passing ultraviolet light (I line 365) through the chrome mask designed by AutoCAD (Autodesk, Inc.). Subsequently, the sample was soft baked at 95 °C for 4 min to remove solvent and to improve adhesion. After post exposure bake at 95 °C for 5 min and developing (SU8 developer, Microchem Corp.), the resulting wafer was coated with FDTS1 (Molecular Vapor Deposition, Applied MicroStructures, San Jose, CA) to promote hydrophobicity of the surface and inhibit PDMS (polydimethylsiloxane, Dow Corning) adhesion. PDMS replica of the channels was made by pouring the degassed mixture of the PDMS oligomer and cross-linker in a ratio of 10:1 w/w onto the patterned wafer. After curing for 4 h at 75 °C, the PDMS was peeled off and punched with biopsy puncher (Miltex) to create access ports. The PDMS slab and the microscope glass were bonded to each other by treatment with oxygen plasma for 1 min under 100 W power (March Jupiter III), and the device was baked at 65 °C for 2 h to enhance bonding. To prevent wetting of the channels, the device was flushed with Aquapel (PPG Industries) followed by drying with nitrogen.

### Microgel droplet generation

Alginate microgels were fabricated by emulsion of the 0.4 wt% alginate-calcium-EDTA mixture in perfluorinated carbon oil (3 M Novec 7500 Engineered Fluid, 3 M, St. Paul, MN, USA) at flow–focusing junction of a microfluidic device. Fluorinated surfactant (Krytox 157 FSH, Dupont) was dissolved in oil (1.75 wt%) to stabilize the droplets. To preserve the spherical shape of the microgel droplets and prevent drop coalescence during gelation out of device, gelation was initiated further downstream of flow–focusing junction by merging of the acidic oil (2 µl acetic acid/ml oil supplemented with surfactant) (Sigma-Aldrich) to the main stream. The acidic environment will release the chelated Ca^2+^ in EDTA allowing it to cross-link the alginate droplet.

The fluids were injected into the microfluidics via polyethylene tubing with inner diameter 0.38 mm and outer diameter 1.09 mm, and 27G × 1/4 needles connected to 1 ml (for alginate solution) and 5 ml (for oil and acidic oil) plastic syringes (all from Becton Dickinson). The syringes were mounted on the syringe pump (PHD 2000, Harvard Apparatus, Holliston, MA, USA), and the fluid were pumped with the flow rate of 50 µl hr^−1^ based on the 1 ml syringe. High-speed camera (Phantom Miro eX4 Monochrome, Vision Research, Wayne, NJ, USA) mounted on inverted microscope (Micro-Spectroscopy Workstation, Zeiss) was used to monitor the droplet generation.

After collection of the microgel droplets in a centrifuge tube, the tube was initially rotated gently back and forth to uniformly mix the acid in the oil to accelerate its diffusion. Then, the tube was placed on rotary plate shaker for 45 min (equal to gelation time) to complete gelation. The oil phase was aspirated off, and 1.5 ml of 20% v/v of PFO (1H,1H,2H,2H-perfluoro-1-octanol 97 %, Sigma-Aldrich) in Novec 7500 oil was added for 3 h to break the emulsions. After remove of oil/PFO phase, the droplets were washed several times with culture medium (centrifuging for 8 min at 94 g). Before final washing, the droplets were placed under ultraviolet light for 30 min for sterilization.

### Alginate concentration regimes

To identify relevant concentration regimes and to characterize the polymer, the RGD-modified alginate was dissolved in deionized water at several concentrations. Steady shear viscosity was measured for compositions ranging from 0.001 to 5 wt% (Supplementary Fig. 3e, f) using a microfluidic device (mVROC, RheoSense Inc), where the viscosity was obtained from pressure drop and flow rate measurements across the microfluidic chip. The concentration of alginate used to make the hydrogels, 0.4 wt%, is in the semi-dilute regime where polymers overlap. This is presumably important to successfully form the polymer networks through inter-chain (rather than intra-chain) crosslinks. At low concentrations, an intrinsic viscosity and associated constant hydrodynamic radius could not be measured. We attribute this to the alginate being a polyelectrolyte^50^ for which the hydrodynamic radius is a function of polymer concentration at sufficiently low salt concentrations. Thus viscosity initially increases sub-linearly as a function of concentration (existing theories suggest a power law of 0.5 in this regime^51^, whereas we observed a power law of 0.64; variance weighted best fit for *c* = 0.01–0.2 wt%). At higher polymer concentrations, our results show a power law of 1.5 (best fit for *c* = 0.8–5 wt%), consistent with existing theories^52^.

### Mechanical characterization of alginate hydrogels

The linear viscoelasticity of alginate hydrogels was probed with a torque controlled rotational rheometer (model AR-G2, TA instruments) using 20 mm stainless steel parallel plate geometry on temperature controlled Peltier system at 25 °C. Alginate disks were prepared by adding alginate-calcium-EDTA solution in custom-made latex rubber molds (~3.5 mm thick) (catalog no. 1374N35, McMaster) adhered to hydrophobic surface followed by addition of acetic acid (12.73 µl acetic acid/ml alginate solution, the same ratio used for droplet gelation). After 4 h of gelation, rubber molds were cut and removed, and cylindrical gel samples were kept in deionized water from 2 to 36 days. Alginate disks were transferred to the rheometer with a 60 grit, adhesive-back sand paper (Norton Abrasives) adhered to the contact surfaces to prevent slipping. To ensure contact initiation, the head was slowly lowered until reaching the normal force of ~30 mN. The disks were trimmed to the size of the flat plate and coated with mineral oil on the exposed edge to minimize solvent evaporation. Strain sweeps were performed to determine linear stress-strain regimes. Subsequently, the frequency sweeps at 1% strain was performed to probe time-dependent mechanical properties. For each concentration, six repeat experiments were performed. To probe the rheology in the nonlinear regime, a separated motor-transducer rheometer (MCR702 from Anton Paar) was used. Raw data were analyzed in MITLAOS^52^ software to obtain Lissajous curves.

### Anchoring alginate hydrogels to glass surface

Alginate was anchored to the glass surface via EDC–Sulfo-NHS chemistry^53,54^. Briefly, microscope slides were degreased with isopropanol and water and dried with nitrogen gas. Then, the glasses were immersed in Piranha solution (H_2_SO_4_ (95–98%): H_2_O_2_ (30%) = 3:1 volume ratio) for 30 min to expose their hydroxyl groups. Subsequently, the glasses were washed with deionized water and covered with 3-aminopropyltriethoxysilane (“APTES”, Sigma Aldrich) for 2 h at room temperature. Amino-silane treated glasses were washed thoroughly with deionized water and dried with nitrogen gas. Then anchoring solution, 1 wt% alginate solution in MES buffer (0.1 M MES and 50 mM NaCl, pH 6.5) with subsequent addition of Sulfo-NHS (molar ratio of 30:1 to alginate), and EDC (molar ratio of 25:1 to alginate), were placed on the functionalized glasses and incubated for 24 h at room temperature.

### Quantitation of hydrogel Poisson ratio

Alginate Poisson ratio was measured using biaxial stretcher (Microscope-Mountable Biaxial Stretching System STB-190-XY, Strex, Inc.). Thin PDMS layer of stretch chamber (STB-VH-04, Strex, Inc.) was removed, and treated sliced microscope slides were glued to opposite sides of the chamber. The chamber was then placed on a petri dish, and alginate-calcium-EDTA solution containing 8.75 μm carboxylated ferromagnetic microparticles (2.72 × 10^6^ particles/ml, Spherotech) was deposited into the chamber and allowed to be gelled for 4 h after the addition of acetic acid. Then, deionized water was added to the chamber to keep the gel hydrated. After two days, the casted gel was cut into thin strips to be free from the chamber side walls while suspended between the glasses. The chamber was placed on the stretcher mounted on the microscope (Leica DM IRE2) followed by stretching the sample to remove any sag. After capturing the first image with a 5X objective, the hydrogel was stretched 10% and imaged to be taken as the second image. To allow for any viscoelastic relaxation, the hydrogel was re-equilibrated for 30 min after each stretching and before capturing each image. After adjusting the threshold for two images, microparticle displacements were calculated using ImageJ PIV plugin.

### Transmission electron microscopy

Microgels were stored in deionized water at 4 °C prior preparation for TEM. The water was removed, and equal volume of water and 4% OsO_4_ (Electron Microscopy Sciences) were added to the microgels for 2 h incubation. Subsequently, 1/2 volume of potassium ferrocyanide (Sigma-Aldrich) was added to the solution, and the microgels were then washed 4 times with water. After rinsing with 25% ethanol, the microgels were serially transmitted to absolute ethanol by 10 min incubations in 50, 75, 95, and 100% ethanol solutions. The microgels were then incubated in mixture of ethanol and LR White resin (Electron Microscopy Sciences) (1:1 volume ratio) for 30 min followed by 1 h incubation in pure LR White resin. The resin was removed and the microgels were incubated in fresh resin at 4 °C for 2 days. The resin was then removed, and fresh resin was added to the microgels to be hardened in 80 °C oven overnight. The tip of hardened resin containing the microgels was cut off from Eppendorf tube using wire cutter and epoxy in to stub. The stub was placed at 80 °C oven for further hardening. The sample was then cut with Rheichart Ultracut and put on grid for imaging under 75 KV H600 Hitachi TEM.

### Cell culture

Murine melanoma cell line B16-F1 was obtained from American Type Culture Collection. The cells were culture on plastic rigid dishes coated with 0.1% gelatin and maintained in DMEM supplemented with 10% fetal bovine serum, (Invitrogen), 1 mM sodium pyruvate, 2 mM L-glutamine (Invitrogen), and 1% penicillin penicillin/streptomycin (Lonza) at 37 °C with 5% CO_2_. Cells were passaged every 2–3 days at a ratio of 1:5 using TrypLE (Invitrogen). For multilayer cell experiments, cells were plated at high density on glass-bottomed petri-dish (MaTek) and cultured for two days to form a confluent layer. Then, 30 µl of the ERMGs’ solution (~6 × 10^7^ ERMGs/ml) was added on top of the confluent cell layer to be encapsulated by cells. For visualization of nuclei and F-actin live imaging, DNA dye Hoechst 33342 (Invitrogen) and SiR-actin (Cytoskeleton) was added to the culture medium at the final concentration of 5 and 1 µm, respectively, 1 h before imaging. Cells were randomly allocated to various experimental groups. A sensitive assay recommended for mycoplasma contamination is DAPI-staining and visual inspection by imaging. The cells were counter-stained with DAPI and continually monitored during the course of experiments for mycoplasma contamination.

### Isolation of TRCs in 3D fibrin gels

Salmon fibrinogen and thrombin were purchased from Reagent Proteins (CA, USA). Fibrinogen was diluted into 8 mg ml^−1^ with T7 buffer (pH 7.4, 50 mM Tris, 150 mM NaCl). Cells were detached from 2D rigid dishes; cell density was adjusted to 1 × 10^6^ cells ml^−1^; then 50 μl cell suspension was mixed with 50 μl ERMGs’ solution in 37 °C cell culture incubator for 30 min. We diluted 100 μl cell and microgels solution in 400 μl DMEM medium (containing 10% FBS and antibiotics), which contained 1 × 10^5^ cells ml^−1^. Fibrinogen and cell solution mixture was made by mixing the same volume of fibrinogen solution (500 μl of 8 mg ml^−1^) and cell solution (500 μl of 1 × 10^5^ cells ml^−1^), resulting in 4 mg ml^−1^ fibrinogen (Young’s modulus = 420 Pa) and 5 × 10^4^ cells ml^−1^ in the mixture. A 200-μl cell and fibrinogen mixture was seeded into each well of 35 mm glass-bottomed dish and mixed well with pre-added 20 μl thrombin (100 U ml^−1^). The cell culture plate was then incubated in 37 °C cell culture incubator for 45 min. Finally, 2 ml of DMEM medium containing 10% FBS and antibiotics was added.

### Immunofluorescence imaging

B16 cells in glass bottom petri dish or 3D fibrin gel were fixed with 4% paraformaldehyde (Electron Microscopy Sciences) diluted in HEPES buffer containing calcium for 20 min at room temperature. Cells were then permeabilized with 0.5% Triton X-100 for 15 min followed by treating with 5% blocking donkey serum (Jackson ImmunoResearch Inc.) for >5 h at room temperature. To investigate the presence of focal adhesions, cells were incubated with paxillin primary antibody (Abcam, Catalog no. ab32084, dilution of 1:100) overnight at 4 °C. After several washing steps to remove unbound primary antibody, cells were incubated with secondary antibody (Abcam, Catalog no. ab150080, dilution of 1:200) at room temperature for 4 h followed by extensive washing to remove unbound secondary antibodies.

To determine effects of substrate treatments on focal adhesions, we incubated cells in serum deprived medium (0.1% serum) for at least 24 h. Glass-bottomed dishes were coated overnight with fibronectin (25 μg/ml; Sigma-Aldrich), a thin layer («1 μm) of alginate/RGD conjugates (1 wt%), or a thick layer (~220 µm thickness) of 1.4-kPa alginate/RGD hydrogel that had the same modulus as the microgels. The cells were then seeded on top of these treated 2D surfaces. After staining with anti-paxillin antibody, the thick alginate/RGD hydrogel was peeled off from the glass and flipped onto coverslips for imaging.

### Microinjection of ERMGs into zebrafish embryos

Wild-type zebrafish (purchased from Chinese Zebrafish Resource Center, The Institute of hydrobiology, Chinese Academy of Sciences, Wuhan, China) was maintained and embryos were raised under published standard conditions^55^. Zebrafish embryos were kept in 31 °C incubator at early stages of development^56^. Blastula-stage embryos (~2.25 hpf, hours post-fertilization) were collected and transferred onto 1% modified agarose gel for microinjection. 5 to 10 nl of 200-nm fluorescent nanoparticle-labeled ERMGs’ solution (~1.2 × 10^7^ ERMGs ml^−1^) suspended in distilled water was injected near the embryo’s blastula tissues using a microinjector (ZGP01500, Zgenebio)^57^. Non-filamentous borosilicate glass capillaries needles (1.0 mm in diameter, Sutter Instrument) were used for the microinjection after they were pulled into finer diameters of ~20 μm. Each injection duration was ~0.04 s at 6 psi injection pressure, resulting in a diameter of the injected droplet ~200 μm. After injection, the fish embryos were collected and mixed with equal volume of 1% agarose. The embryos in 0.5% agarose were immediately transferred to 35-mm glass-bottomed dish, and water was added after agarose solidification. We used the needle to puncture the outer membrane of the zygote into water spaces surrounding the embryonic tissues to inject the microgels, but not into the embryonic tissues, so the perturbation caused by the needle was negligible. The injected embryos appeared to develop normally for at least 5 days post fertilization. After finishing experiments, leftover embryos were deeply anesthetized with 0.04 mg ml^−1^ tricaine (MS-222, Sigma) and put on ice to die. All studies that use zebrafish were approved by the Animal Care and Use Committee of School of Life Science and Technology at Huazhong University of Science and Technology. All experimental protocols were in strict accordance with guidelines from the Laboratory Animal Training Association and IACUC protocols.

### Imaging

Samples on glass bottom petri-dish were imaged under laser confocal microscope (Leica TCS SP8) equipped with resonant scanning mirrors for high speed imaging (35 frames/s for 512 × 512 pixels images). Most images were taken using 63× oil-immersion objective with 1.4 NA, however, for some samples especially 3D melanoma colonies, 40× oil-immersion objective with 1.3 NA was used to image thick samples. For 3D confocal volume, *z*-stack were acquired with 0.2–0.3 μm step and 512 × 512 focal planes with a pixel size of ~0.15 μm over a 45–60 s period with line averaging of 3. After acquiring 3D stacks of stressed images, the cells were treated with 3% Triton X-100 detergent or 1 μM Latrunculin A (Sigma-Aldrich) and then time-lapse datasets with time interval of 3–5 min were acquired for 1–2 h until the stress-free condition for the microgel was achieved. It was verified that Triton had no effect on the microgel alone. All samples were imaged under 37 °C and 5% CO_2_ controlled by a mounted incubator chamber (Okolab stage top incubator).

### Traction calculation

Before traction calculation, 3D confocal images were deconvoluted (AutoQuant X3), and the separated free nanoparticles from the microgel were discarded using pencil tool in ImageJ (NIH). This step will enhance the image quality and prevent artifacts in the recovered displacements. Cell-induced displacement fields of embedded fluorescent nanoparticles were obtained by FIDVC technique. This technique can capture the deformation using an iterative deformation method and refining interrogation window size in each iteration. Once the displacement field was determined, cell-induced infinitesimal strain tensor was calculated using displacement gradient tensor (∇**u**), calculated with Optimal-11 tap finite difference kernel.

2 $${\mathbf{E}} = \frac{1}{2}(\nabla {\mathbf{u}} + \nabla {\mathbf{u}}^{\mathrm{T}})$$

Based on the experimentally measured alginate mechanical properties and strain range (<25%), isotropic and linear constitutive model was assumed to calculate the Cauchy stress tensor.

3 $${\mathbf{\sigma }} = 2\mu {\mathbf{E}} + \lambda \mathrm{trace}({\mathbf{E}}){\mathbf{I}},$$

where **σ** and **E** are the Cauchy stress and strain tensor, respectively; *μ* and *λ* are the Lamé parameters. Since some samples in multilayered cells condition showed maximum principal strain of up to 40% in small regions of their surface, the Neo-Hookean model was proposed to estimate the extent of underestimation based on the linear assumption.

4 $${\mathbf{\sigma }} = \frac{\mu }{{J^{\frac{5}{3}}}}({\mathbf{B}} - \frac{1}{3}\mathrm{trace}\left( {\mathbf{B}} \right){\mathbf{I}}) + (\lambda + \frac{2}{3}\mu )(J - 1){\mathbf{I}},$$

Where *J* and **B** are the jacobian of the deformation gradient tensor (**F** = ∇**u**+**I**) and the left Cauchy Green’s tensor, respectively.

5 $$J = \det \left( {\mathbf{F}} \right)$$

6 $${\mathbf{B}} = {\mathbf{F}}.{\mathbf{F}}^{\mathrm{T}}$$

Microgel surface tractions were computed at inner surface (~1 µm offset from the microgel surface) to remove boundary effect on the recovered tractions (Supplementary Fig. 20). Briefly, the surface of the microgels was computed using the concept of convex hull. In this approach, centers of nanoparticles were located, and the point cloud of the nanoparticles was created using their spatial coordinates. Then, the smallest convex envelope of the set of discrete points was calculated using built in convex hull function in MATLAB. The boundary points were then removed for four layers, and the new exposed boundary points were used to create surface mesh using triangulation. Stress tensor at triangle midpoint was used to calculate surface traction:

7 $${\mathbf{T}} = {\mathbf{\sigma }}.{\mathbf{n}}$$

### Resolution and noise analysis

The resolution and noise sensitivity test were conducted using simulated traction field on 1600 Pa elastic spheres of 24-µm in diameter. Forward finite element method was carried out by Abaqus (Dassault Systèmes) using ten-node linear tetrahedron elements “C3D10”. Then, the resulting nodal displacement and coordinates were imported into MATLAB, and the displacement was interpolated into three-dimensional matrix with the same matrix and pixel size of the reference image (Gaussian white noise can be superimposed with the interpolated displacement matrix for noise analysis). The reference image was constructed by creating the randomly positioned fluorescent nanoparticles with similar point spread function as the experimental images and different densities (0.001–0.006 nanoparticles per voxel). Then, the nanoparticles that were out of the 24-µm sphere were removed. The deformed image was created by moving the position of the fluorescent nanoparticles in the reference image with respect to the interpolated displacement at their positions. Finally, the two created images were compared by FIDVC, and simulated traction were calculated using recovered displacement fields.

### Data availability

All relevant data are available from the authors upon request to the corresponding author.

All codes used for computing deformation and traction are available, free of charge, upon request to the corresponding author.

### Statistical analysis

One-tailed Student’s *t*-test was used, followed by Bonferroni comparison for multiple comparisons.

## Electronic supplementary material

Supplementary Information
