# Supplementary material for: Quantifying compressive forces between living cell layers and within tissues using elastic round microgels
Source: Nat Commun. 2018 May 14;9:1878. doi: 10.1038/s41467-018-04245-1 (PMC5951850; doi:10.1038/s41467-018-04245-1)
Supplement: Supplementary file 1 — Supplementary Information [file 41467_2018_4245_MOESM1_ESM.pdf]

Supplementary Information

Quantifying compressive forces between living cell layers and within tissues using elastic round microgels

Mohagheghian *et al.*

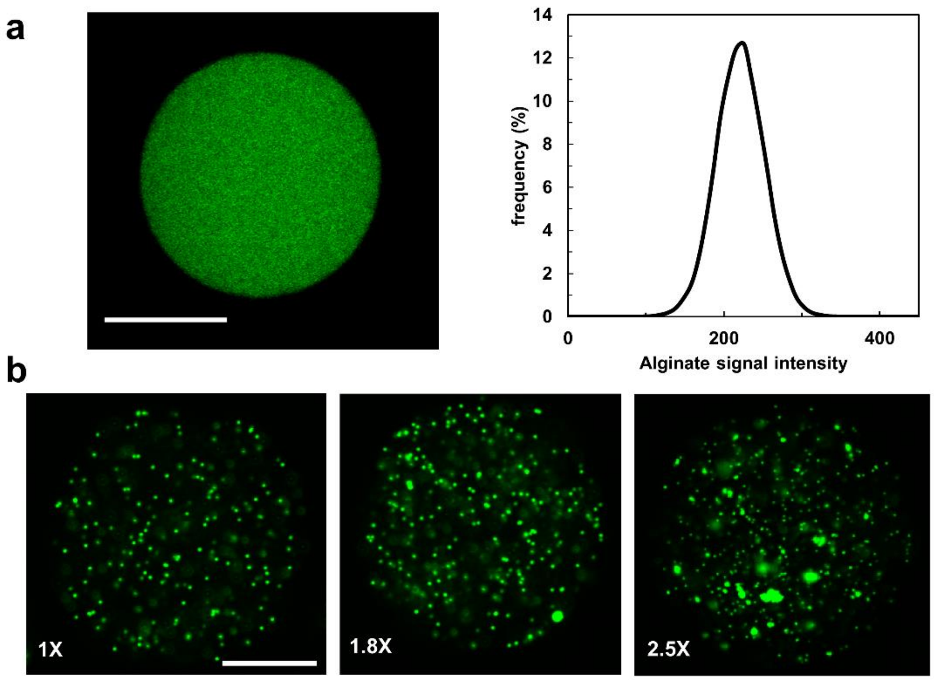

Supplementary Figure 1. Homogeneity of alginate microgels. (a) Left: An image of a FITC-alginate microgel, cross-linked by acidic release of chelated Ca in Ca-EDTA complex due to a drop in pH; **Right**: A single peak of alginate signal intensity indicates the homogenous internal structure verifying the assumption of a homogenous material of the microgel. Scale bar, 15 µm. **(b)** Confocal cross sections of the droplets with different concentrations of fluorescent nanoparticles in the alginate-Ca-EDTA solution. 1X represents a concentration of ~5.7×10^{10} nanoparticles (200 nm in diameter) in 1 ml alginate-calcium-EDTA solution. 1.8X concentration shows an optimal concentration with little aggregation. Scale bar, 10 µm.

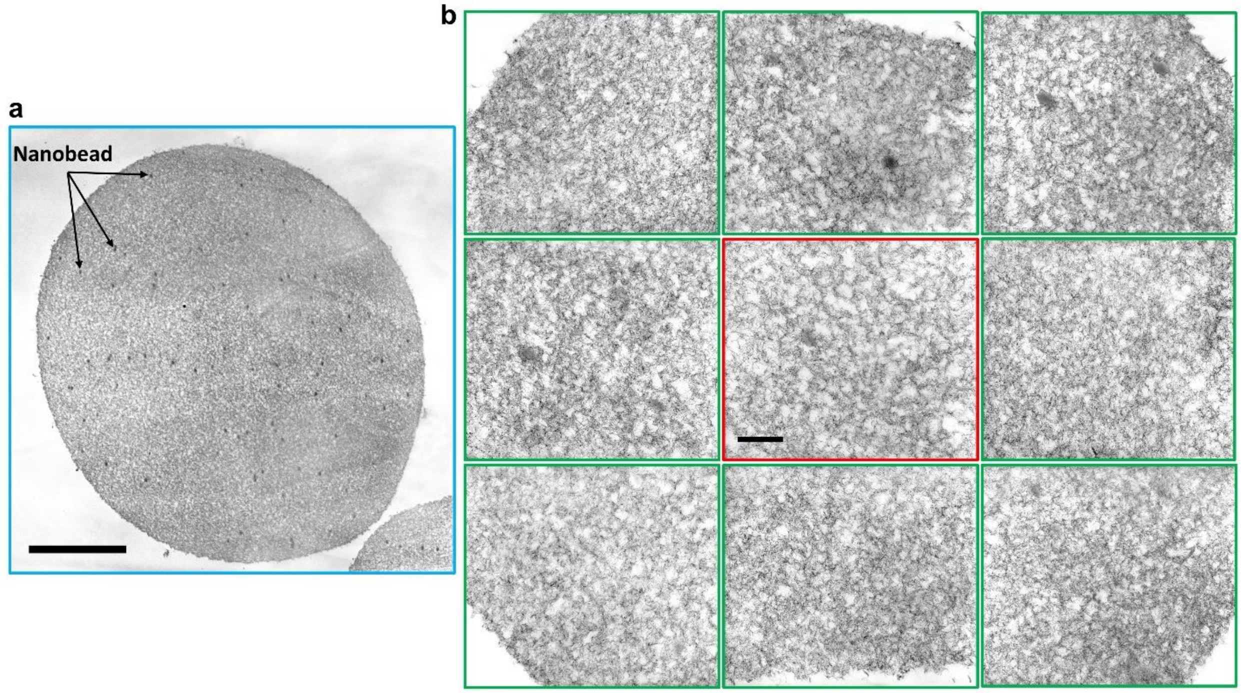

Supplementary Figure 2. Internal structures of alginate microgels are uniform. Transmission electron microscopy (TEM) image of an ERMG with 4000x magnification (**a**) (Scale bar, 5.0 µm)**,** and its corresponding 30000x magnified images of 9 randomly-chosen views (**b**) (Scale bar, 0.5 µm) in clockwise positions including the center.

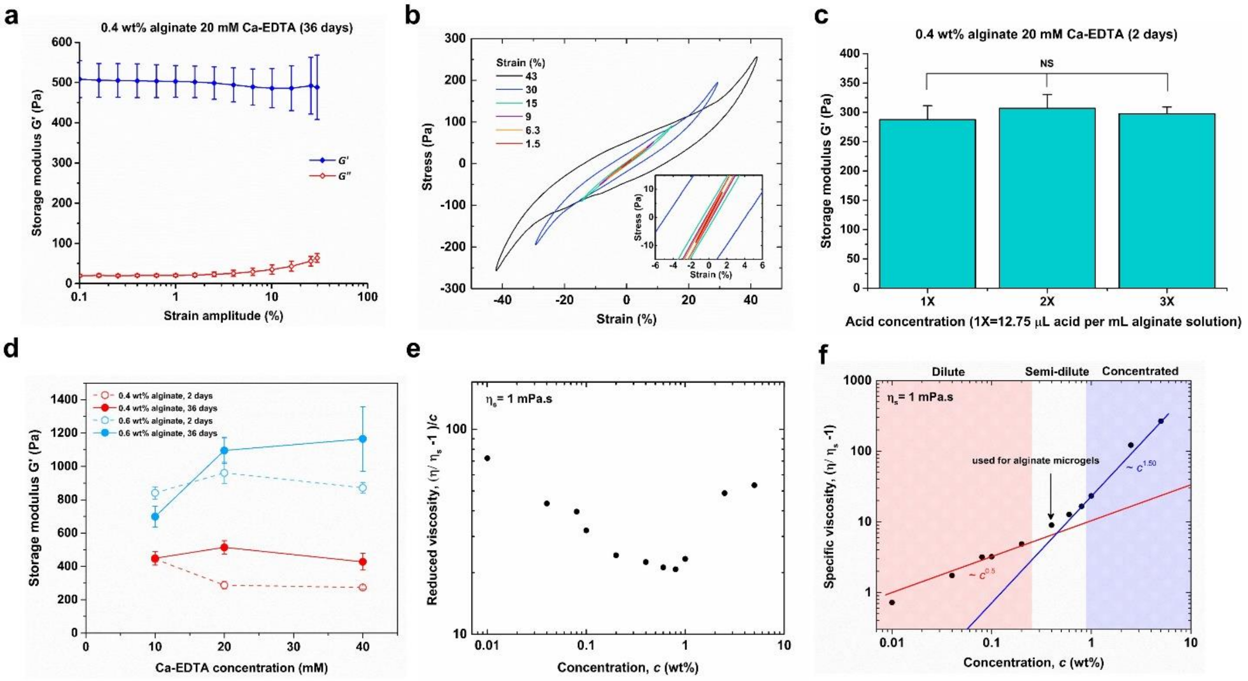

Supplementary Figure 3. Mechanical characterization of alginate hydrogels. (a) Oscillatory strain sweeps of Alginate (0.4 wt%, 20 mM Ca-EDTA) at a frequency ω = 1 rad/s. The first harmonic storage shear modulus is shown as a function of applied strain. **(b)** Raw stress-strain data (Lissajous curves) reveal nonlinear characteristics of the material: at strains >14%, shapes of Lissajous curves deviate from elliptic shapes (linear viscoelastic), indicating intra-cycle strain stiffening in the alginate hydrogel. However, the variation of the mean modulus at strains up to 30% is still within the range of error bars, justifying the linear assumption for this range of strain. **(c)** Average storage shear modulus of alginate as a function of volume of acetic acid added to initiate gelation. Storage modulus showed no significant dependence for the range of acid volume used for microgels' gelation. NS, not statistically significant. One-tailed Student's t-test was used, followed by Bonferroni comparison. **(d)** Effect of hydration time, alginate and Ca-EDTA concentration on the storage modulus of the alginate hydrogels. ERMGs that are used in living cell/tissue experiments are made of 0.4% alginate with 10 or 20 mM Ca-EDTA. **(e)** The reduced viscosity of solution of Sodium Alginate in water plotted as a function of polymer concentration. For simple polymer solutions, the reduced viscosity approaches a constant value as $c \rightarrow 0$ (defined as intrinsic viscosity). Sodium Alginate being a polyelectrolyte does not follow such a trend[1]. As shown in the figure reduced viscosity increases in the limit $c \rightarrow 0$. **(f)** The viscosity measurements are consistent with the Fouss's Law with specific viscosity increasing as $c^{0.5}$ (red line) at dilute concentrations (variance weighted fit value of the power-law is 0.64±0.03). At high concentrations, the specific viscosity increases as $c^{1.5}$ (blue line) as reported in literature[2] (fit value 1.51±0.07). Hence, the Sodium Alginate concentration of 0.4 wt% used to form microgels falls in the semi-dilute regime. Mean ± s.e.m., n = 6 samples each for (a), (c), and (d).

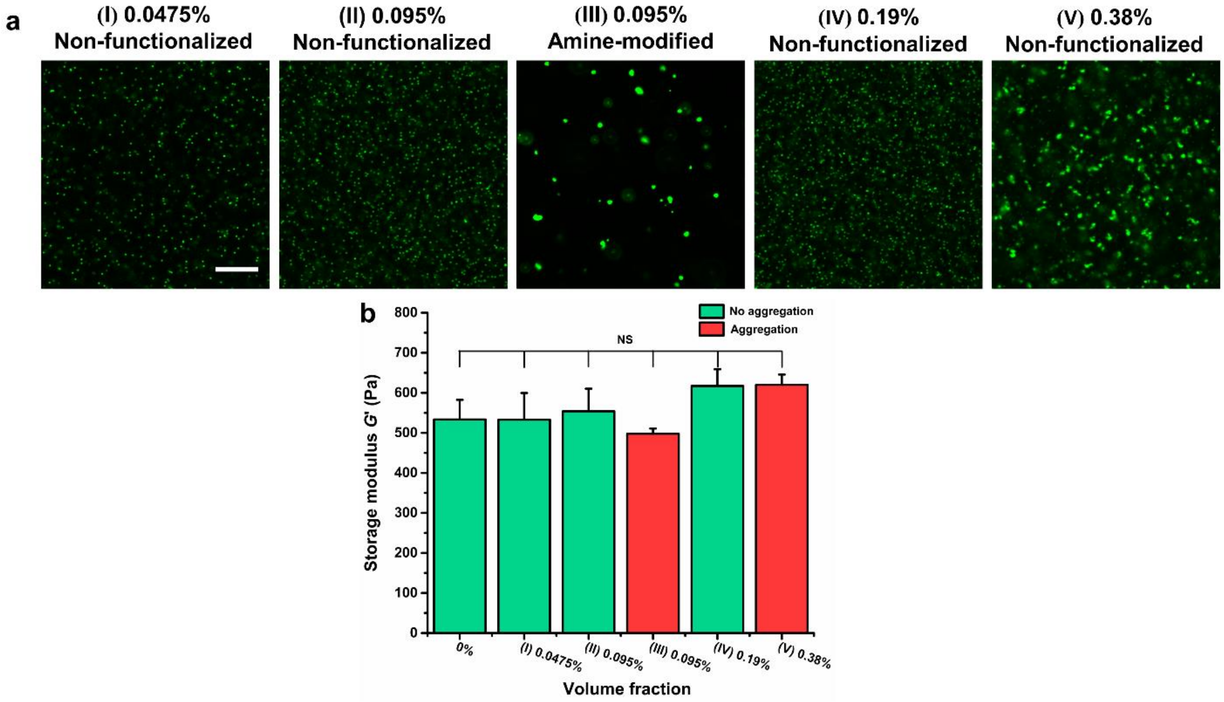

Supplementary Figure 4. Volume fraction and aggregation effects of nanoparticles on storage modulus of the alginate hydrogels. (a) Confocal cross sections of the hydrogel samples used for rheology experiments. For the middle image, amine-modified fluorescent nanoparticles were used to study aggregation of nanoparticles with the same volume fraction. Scale bar, 10 µm. **(b)** Storage modulus showed no significant dependence for the range of nanoparticle volume fraction used for microgels' gelation. NS, not statistically significant. Mean $\pm$ s.e.m., $n = 4$ samples. One-tailed Student's t-test was used, followed by Bonferroni comparison.

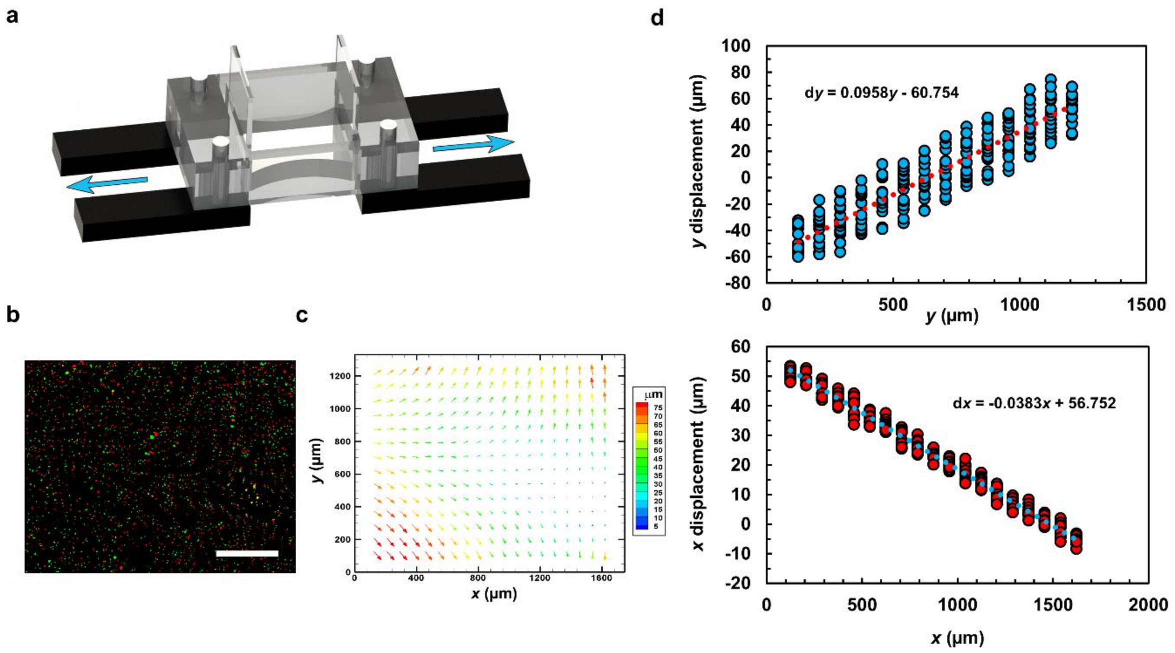

Supplementary Figure 5. Determination of alginate hydrogel Poisson ratio. (**a**) 3D drawing of a stretcher setup with a mounted chamber and hydrogel suspended between treated glasses. (**b**) Overlay of images of 8.75-µm ferromagnetic microparticles embedded in an alginate hydrogel before (green) and after (red) 10% stretch. Scale bar, 400 µm. (**c**) Corresponding stretch-induced displacement fields. **(d)** Scatter plot of *x* and y displacements as a function of *x* (bottom panel) and *y* (top panel) positions, with a linear fit to each dataset. The Poisson ratio is calculated as the negative ratio of slopes of linear fits, - (d*x*/*x*) divided by (d*y*/*y*), and equals 0.4. Assuming an isotropic material based on the uniform distribution of alginate in the alginate hydrogels, the Poisson ratio is estimated to be 0.4 in all directions.

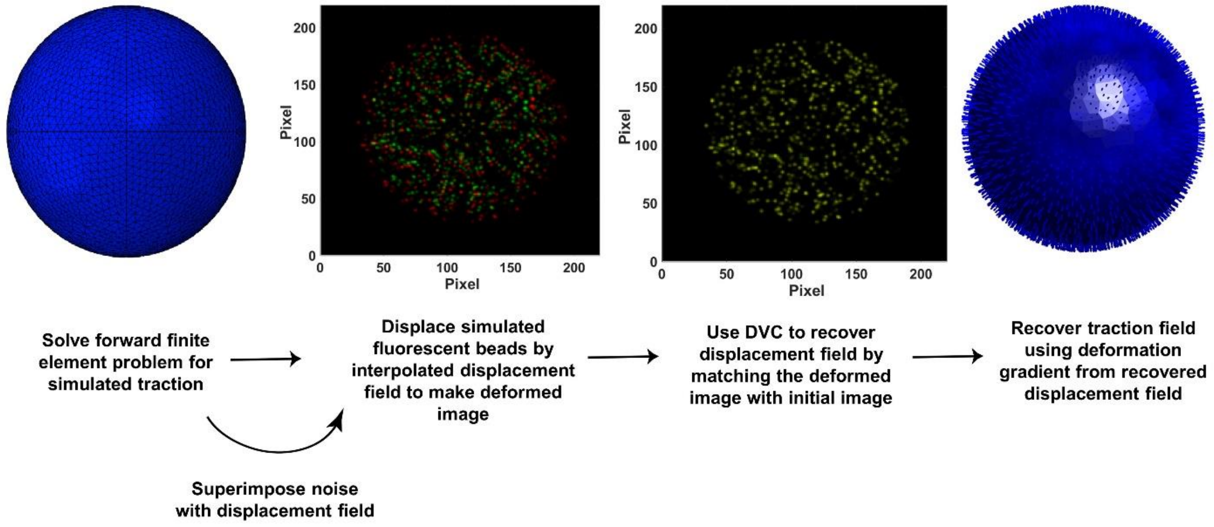

Supplementary Figure 6. Flowchart of noise analysis using simulated traction field. Using ABAQUS software**,** forward finite element analysis was solved for a given traction to calculate nodal displacements. Then, nodal coordinates and displacements were exported into MATLAB to calculate interpolated displacement fields in the form of three dimensional matrices with the size of initial images (Gaussian white noise are superimposed with the interpolated displacement matrix for noise analysis). Next, simulated fluorescent nanoparticles (with similar point spread function (PSF) in the actual cell experiments) in the initial image were translated with respect to the interpolated displacement fields to create deformed images (red: before deformation, green: after deformation). Using digital volume correlation, the initial and deformed images were compared, and the displacements were recovered. Finally, the applied traction was recovered by calculation of deformation gradients and application of elasticity equations.

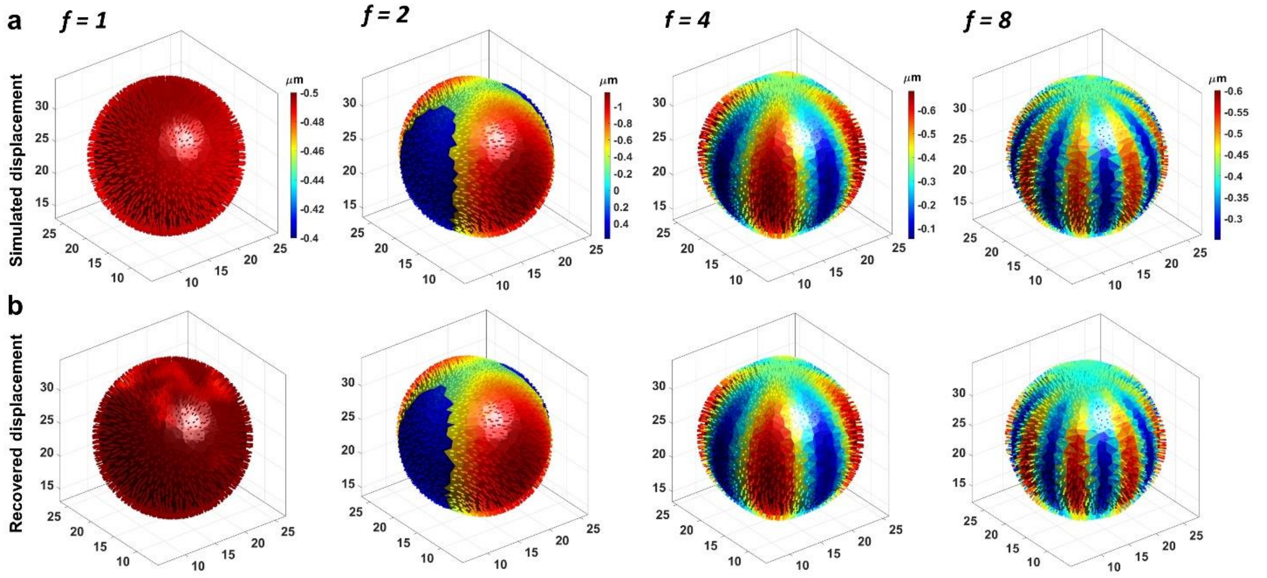

Supplementary Figure 7. Resolution analysis using simulated deformation. (a) 3D Contour plots showing the magnitude of simulated displacements. (**b**) Corresponding recovered displacement field on the surface of 24-μm sphere (Young's modulus=1600 Pa) with four different modes of compression (left panel, uniform (*f*=1). **Middle left panel**, the sphere was compressed periodically in half (*f*=2); **middle right panel**, the sphere was compressed periodically in quarter (*f*=4); **right panel**, the sphere was compressed periodically in eighth (*f*=8).

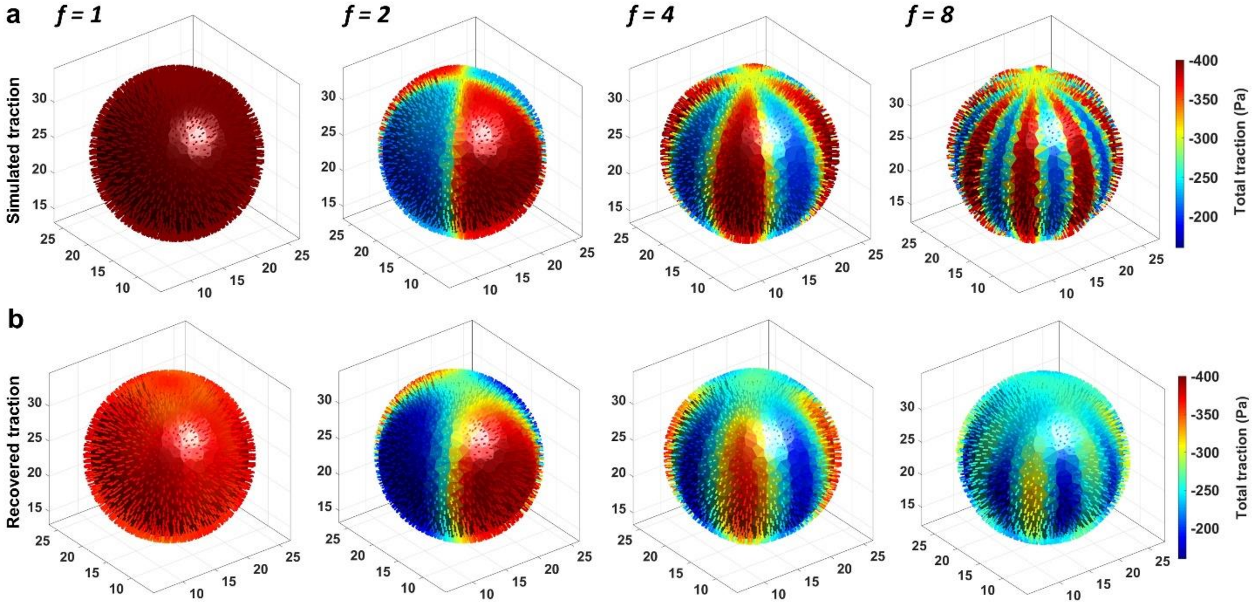

Supplementary Figure 8. Resolution analysis using simulated tractions. (a) 3D Contour plots showing the magnitude of simulated tractions. (**b**) Corresponding recovered traction fields on the surface of 24-µm sphere. Compressive loadings were applied using similar modes as Supplementary Fig. 7.

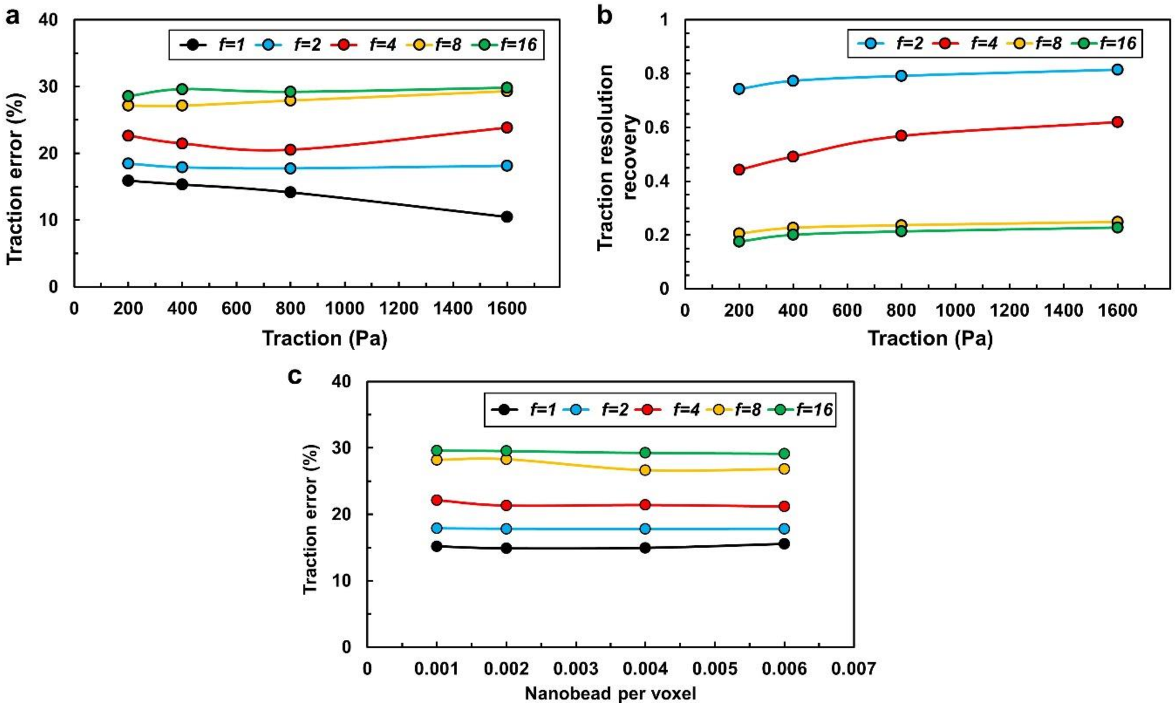

Supplementary Figure 9. Resolution analysis using simulated tractions. (a) Percent traction error averaged over all faces of the microsphere as a function of traction amplitude and periodicity over the microsphere surface. **(b)** Traction resolution recovery (equation (1)) as a function of traction amplitude and periodicity over the microsphere surface. **(c)** Percent traction error as a function of nanobeads density and traction periodicity for applied compression of 400/200 Pa (0.003 nanobead per voxel ≅ 0.095% nanoparticle volume fraction).

$$\text{Traction resolution recovery} = \frac{|standard\ deviation\ of\ recovered\ tractions\ for\ all\ faces|}{|standard\ deviation\ of\ simulated\ tractions\ for\ all\ faces|} \tag{1}$$

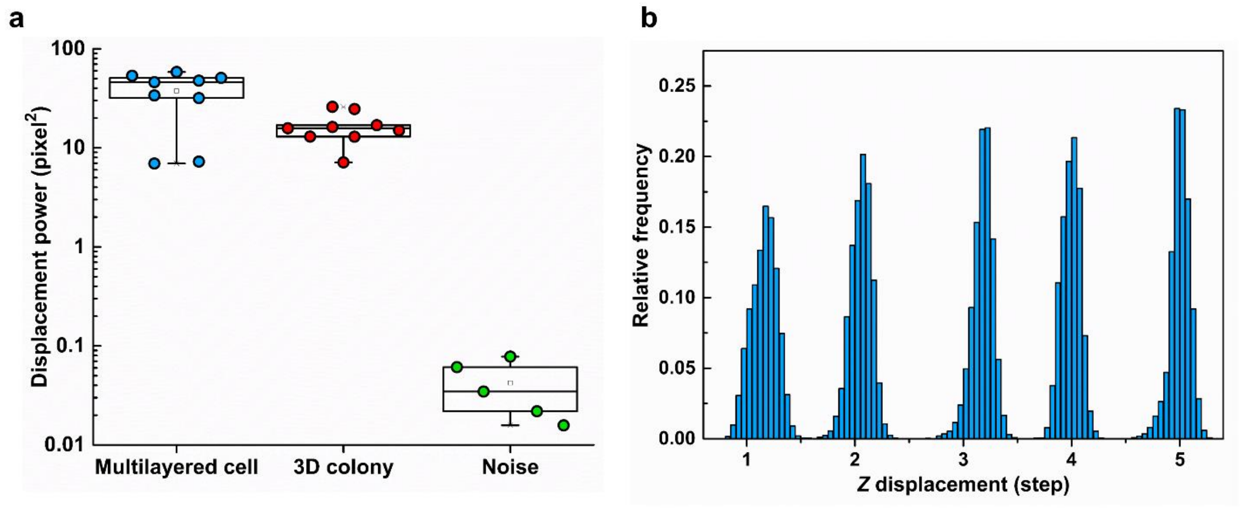

Supplementary Figure 10. Noise and tracking analyses of the microgel images. (a) Comparison of displacement power (mean square of the displacement field) for the deformed microgels in multilayered cells and 3D colony with respect to the noise level. The noise power was measured by imaging the isolated microgels in fibrin gel without any treatments and without any nearby cells. Solid lines in the box plots correspond to 25th percentile, median and 75th percentile. **(b)** Histograms of *z* displacements for the microgel experimentally translated 5 steps (=1 µm) at 0.2-µm intervals. The fast iterative digital volume correlation (FIDVC) is able to quantify 0.2-µm increments in the axial direction.

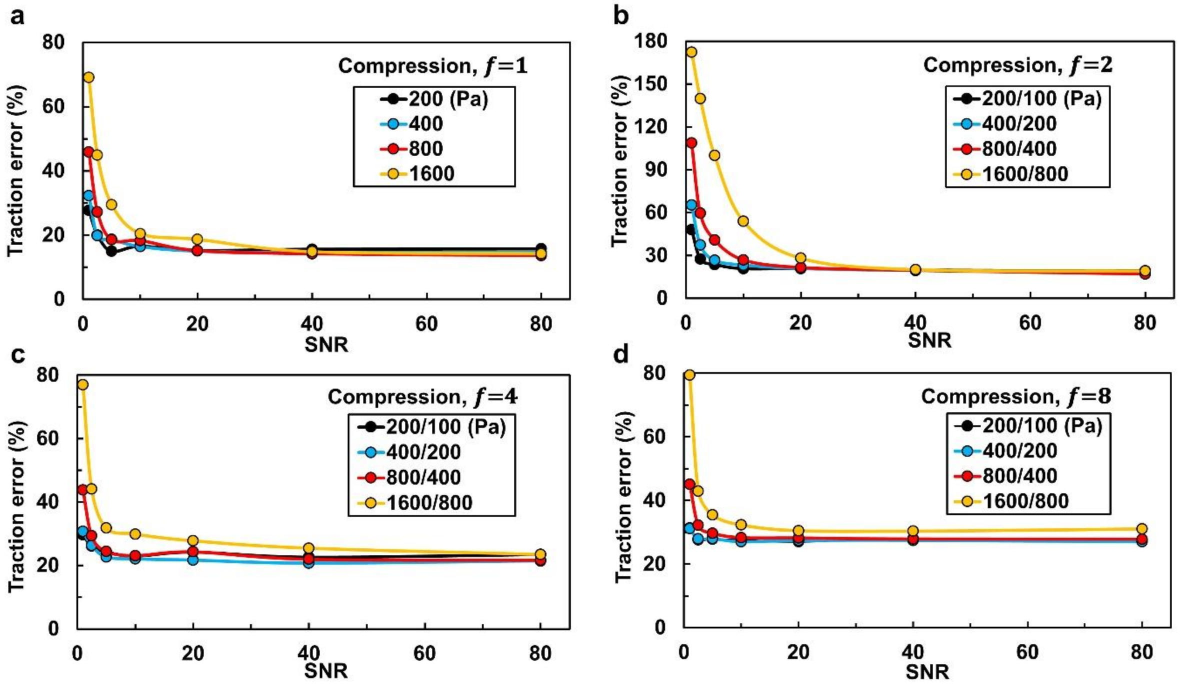

Supplementary Figure 11. Noise and sensitivity analyses using simulated tractions. (a-d) Plots of averaged percent traction error as a function of traction amplitudes and signal to noise ratio for periodic compressive traction with different loading wavelengths (D, diameter of sphere=24 µm; *f*, number of loading repetitions; λ=πD/*f*=75.4 µm for *f*=1; l=37.7 µm for *f*=2; λ=18.85 µm for *f*=4; l=9.43 µm for *f*=8). Different loading wavelengths are a measure of sensitivity of the ERMG method on tractions. Typical experimental SNRs are ≥ 1000 and thus traction errors are ~25% or less in different loading conditions.

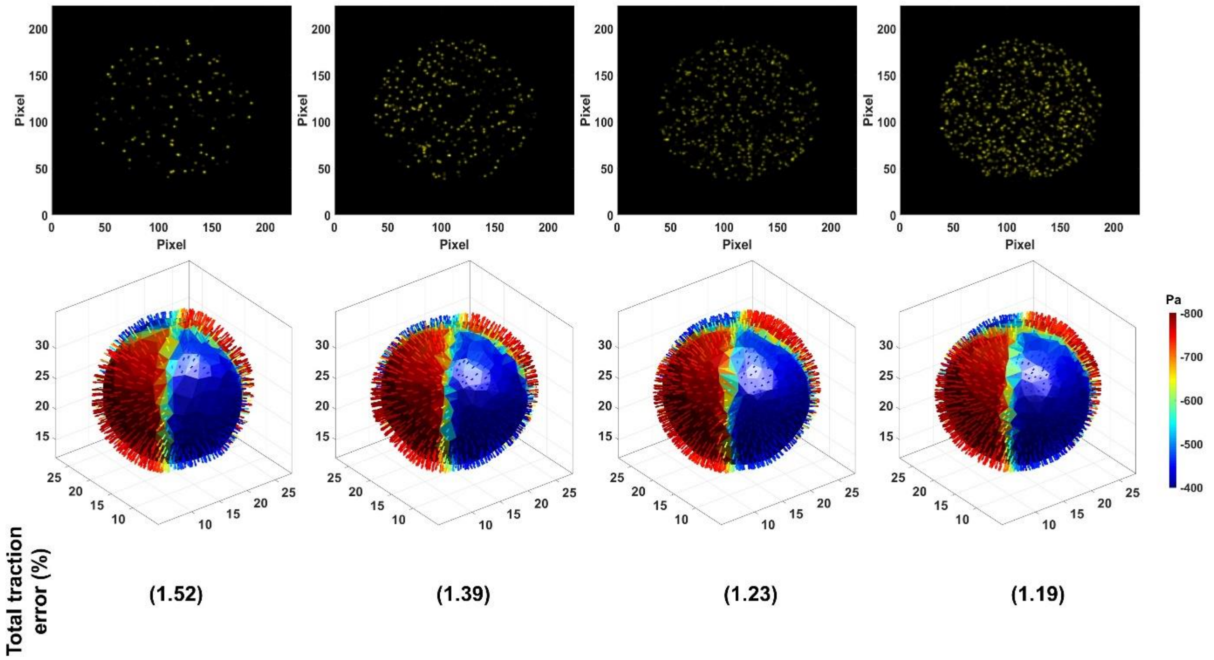

Supplementary Figure 12. Traction recovery sensitivity with respect to microgel surfaces. Top, simulated images with different nanoparticle density to generate the sphere surfaces with different coarseness and normal vectors. **Middle,** 3D simulated traction calculated over its corresponding generated surface from the top panel image. **Bottom**, mean normal traction error with respect to the surface generation with different coarsenesses. For all surface meshes, the error is calculated as the difference between the calculated traction vector and the actual traction vector from the precise normal vector. The actual normal vector is known as the gradient of the sphere at the location of the surface mesh (equation (2)).

$$\nabla = \frac{(x, y, z)}{\|(x, y, z)\|} \tag{2}$$

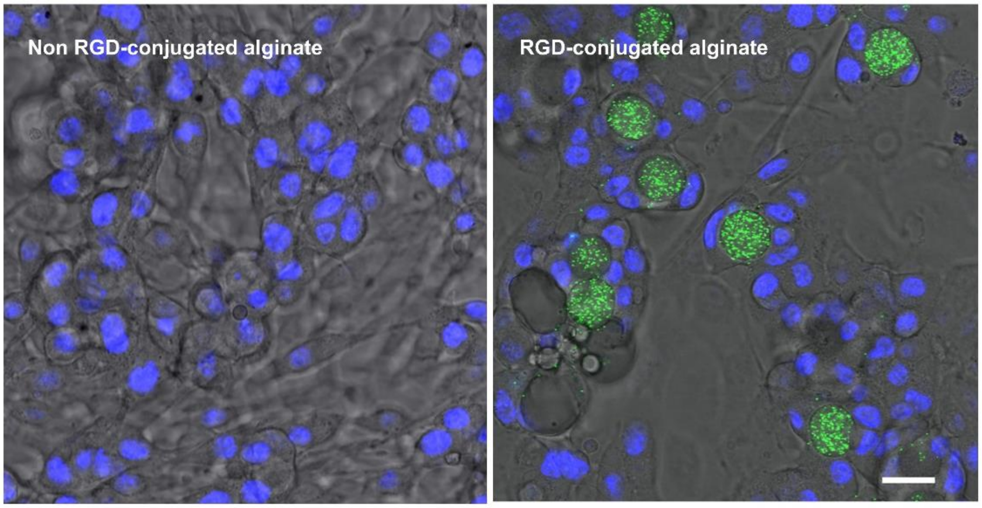

Supplementary Figure 13. Cells attach to RGD-conjugated alginate microgels. ERMGs were fabricated from RGD-conjugated alginate or non RGD-conjugated alginate. RGD-conjugated alginate microgels (green colors, right image) were attached to and encapsulated by the cells (blue colors are DAPI staining of cell nuclei), but un-functionalized microgels that were added to the cells (left image) did not bind to the cells and were easily washed away with fresh medium. Scale bar, 25 μm.

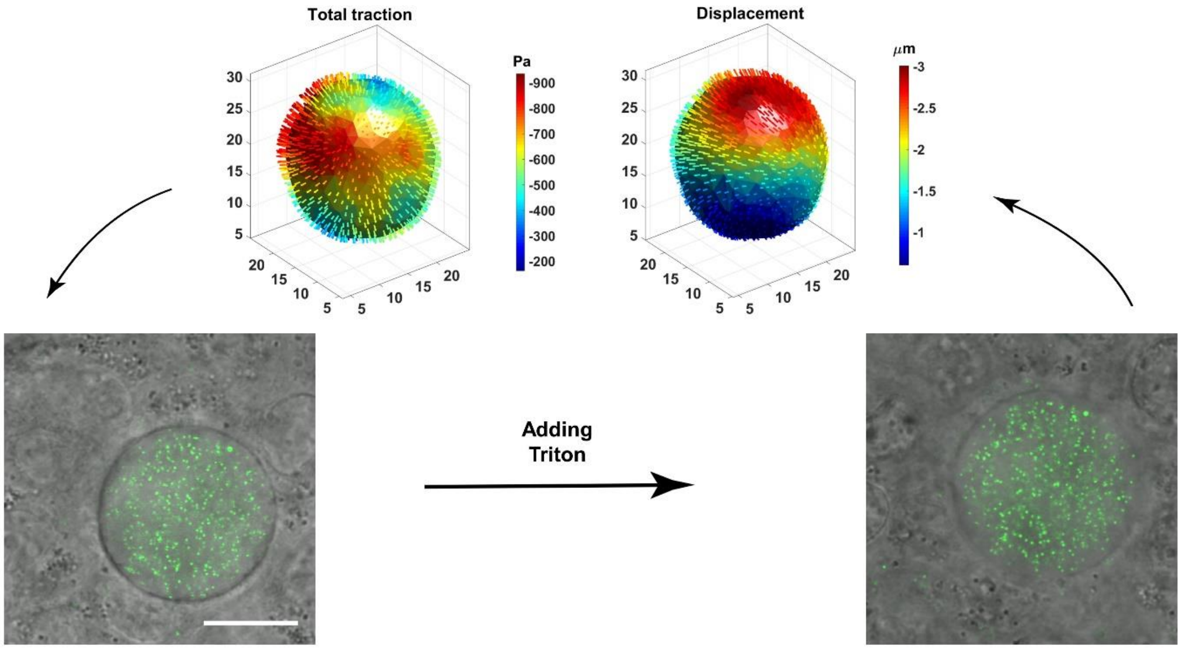

Supplementary Figure 14. A compressed embedded microgel inside a melanoma colony. Bottom, a confocal section of an embedded ERM in the colony before and after addition of Triton X-100 (3%). Scale bar, 15 µm. **Top**, corresponding 3D displacements and total traction showing the compressed state of the microgel.

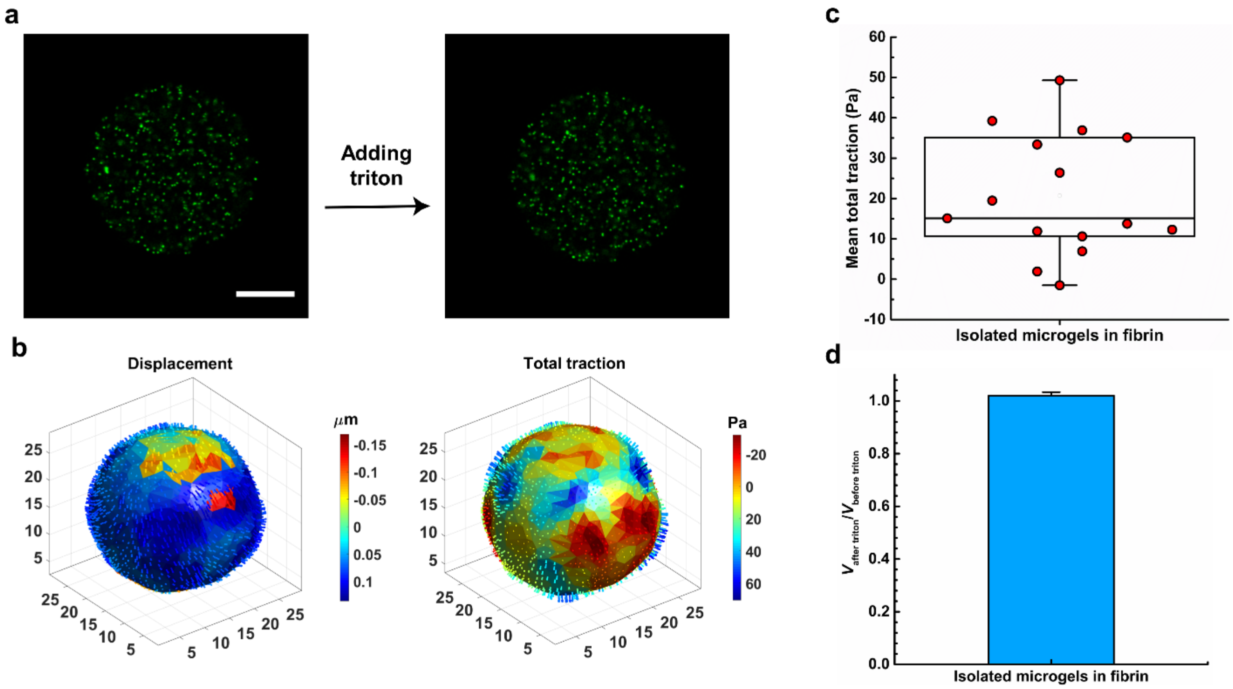

Supplementary Figure 15. Triton effect on the microgels. (a) Representative confocal fluorescent images of the isolated microgel in a fibrin gel before and after addition of Triton X-100 (3%). Scale bar, 10 µm. **(b)** Corresponding 3D displacements and total traction of the microgel treated with Triton. **(c)** Scatter plot of mean total traction calculated over 15 isolated microgels in the fibrin gel treated with Triton. Solid lines in the box plots correspond to 25th percentile, median and 75th percentile. **(d)** Triton treatment has no significant effects on the volume of the microgel. Mean ± s.d.m., *n*=15 microgels.

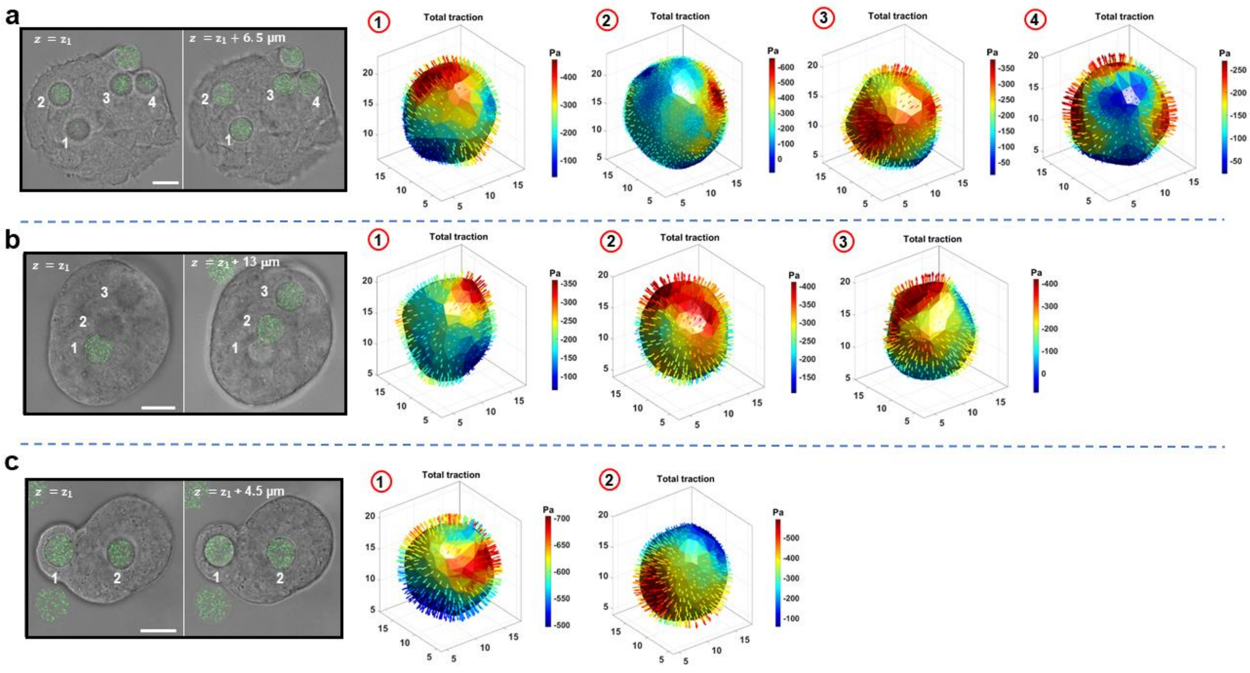

Supplementary Figure 16. Multiple microgels inside a melanoma cell colony. (a) Four microgels are encapsulated in one colony (**Left**, at a focal plane; **right**, at a different focal plane). Tractions of a microgel on the top of the colony could not be quantified. **(b)** Three microgels are encapsulated in one colony (**Left**, at a focal plane; **right**, at a different focal plane). (**c**) Two microgels are encapsulated in one colony (**Left**, at a focal plane; **right**, at a different focal plane). All scale bars, 20 μm. Corresponding 3D total tractions were computed from deformations of these microgels, referenced to Triton-treated unstressed condition. For clarity, only total tractions are shown here. Note that substantial differences exist in traction distribution among various microgels within a given colony.

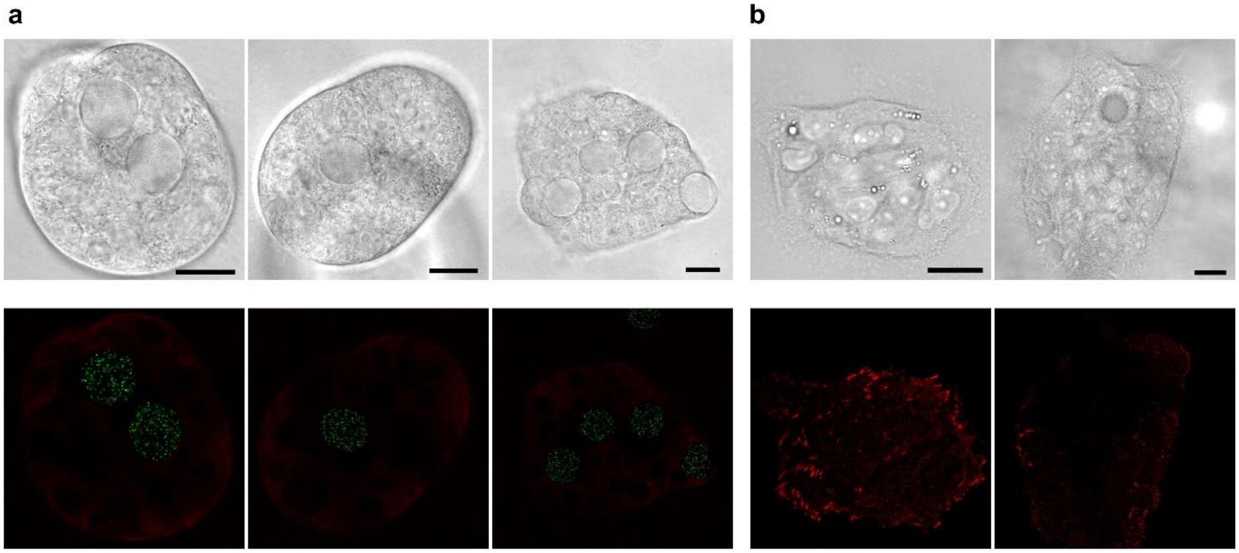

Supplementary Figure 17. No focal adhesions near the microgel inside a melanoma cell colony. (a) Representative brightfield images of 3 different melanoma colonies (**top**) and their corresponding merged fluorescent images (**bottom**) of paxillin (**red**) and the embedded microgels (**green**) ~10 μm above the glass. **(b)** Brightfield (**top**) and the corresponding fluorescent (**bottom; red=paxillin**) images of two different melanoma colonies just above the glass surface. All samples were imaged from the same dish. All scale bars, 20 μm. Note that no focal adhesions were present between colonies and its microgels in 3D gels except for colonies just above the glass in subfigure (**b**).

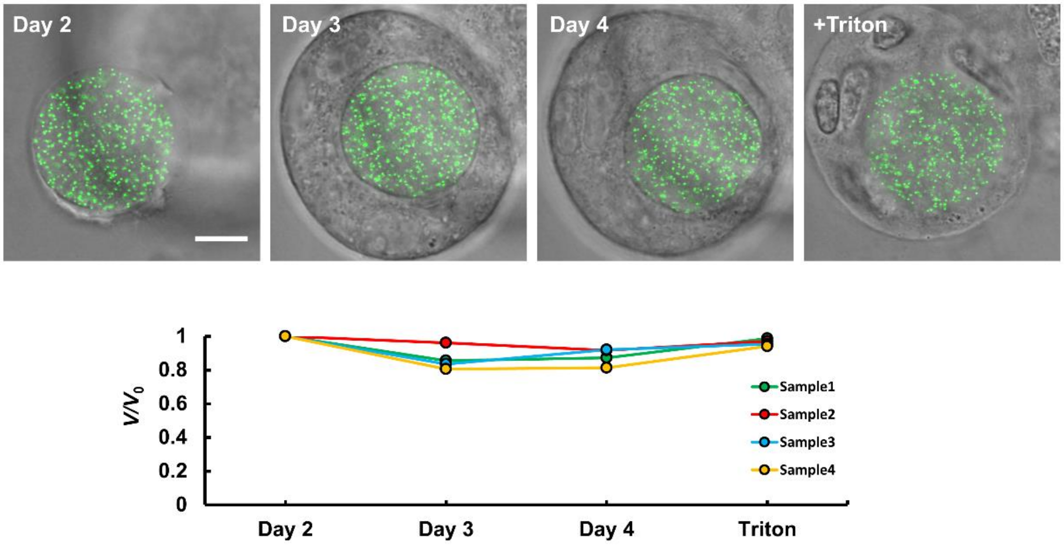

Supplementary Figure 18. No degradation and volume loss for ERMGs trapped in cells. Top, representative confocal fluorescent images showing the encapsulation of an ERMG without any diffused out nanobeads. Scale bar, 10 µm. **Bottom,** volume change of 4 ERMGs with respect to their initial volume (V_0) at day 2. The microgels were just attaching the cells at day 2, but they were encapsulated inside a colony at day 3 and day 4. Microgel volume reduction at day 3 and day 4 is due to compressive stresses by surrounding cells. Sample 1 is corresponding to the microgel in the top panel. No changes of microgel volumes when comparing day 2 with after Triton treatment indicate no degradation and volume loss of the ERMGs by cells and Triton.

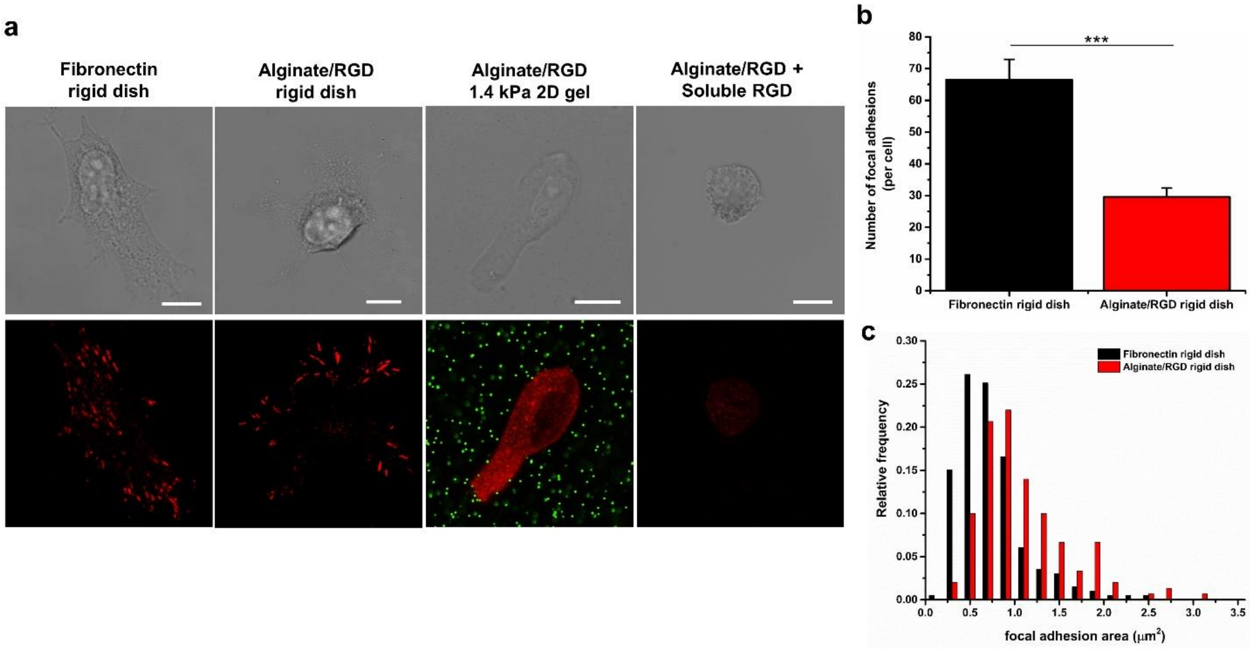

Supplementary Figure 19. Stiffness of alginate-RGD coated 2D surface determines formation of focal adhesions. (a) Representative brightfield images of B16-F1 cells on different substrates (**top**) and their corresponding fluorescent images (**bottom**) of paxillin (**red**) and embedded fluorescent nanobeads (**green**) in 1.4-kPa alginate gels. From left to right: fibronectin (25 μg per ml) coated rigid glass, a thin layer (<<1 μm) of alginate/RGD (1 wt%) coated glass, a thick layer (~220 μm) of 1.4-kPa alginate/RGD hydrogel coated glass, and alginate/RGD coated rigid glass plus 50 μg/ml soluble RGD. All fluorescent images were background-noise subtracted and processed with the same contrast. All scale bars, 10 μm. Note that the cells on top of 1.4-kPa alginate/RGD hydrogel formed no mature (and elongated) focal adhesions but generated dotted and rounded focal plagues/complexes. Soluble RGD addition led to cell rounding and loss of focal adhesions, in addition to dramatic reduction of the number of adherent cells, suggesting that these cells adhered to alginate/RGD coated surfaces specifically via RGD. **(b)** Number of focal adhesions of cells cultured on rigid dishes with different surface treatments. Mean ± s.e.m.; n=19 cells for each condition; ***P <0.001. One-tailed Student's t-test was used. **(c)** Comparison between fibronectin and alginate/RGD on focal adhesions size distribution. n = 200 focal adhesions for each condition. Cells on alginate/RGD coated 2D rigid planar surface generate fewer but larger mature focal adhesions than those on fibronectin coated rigid surface.

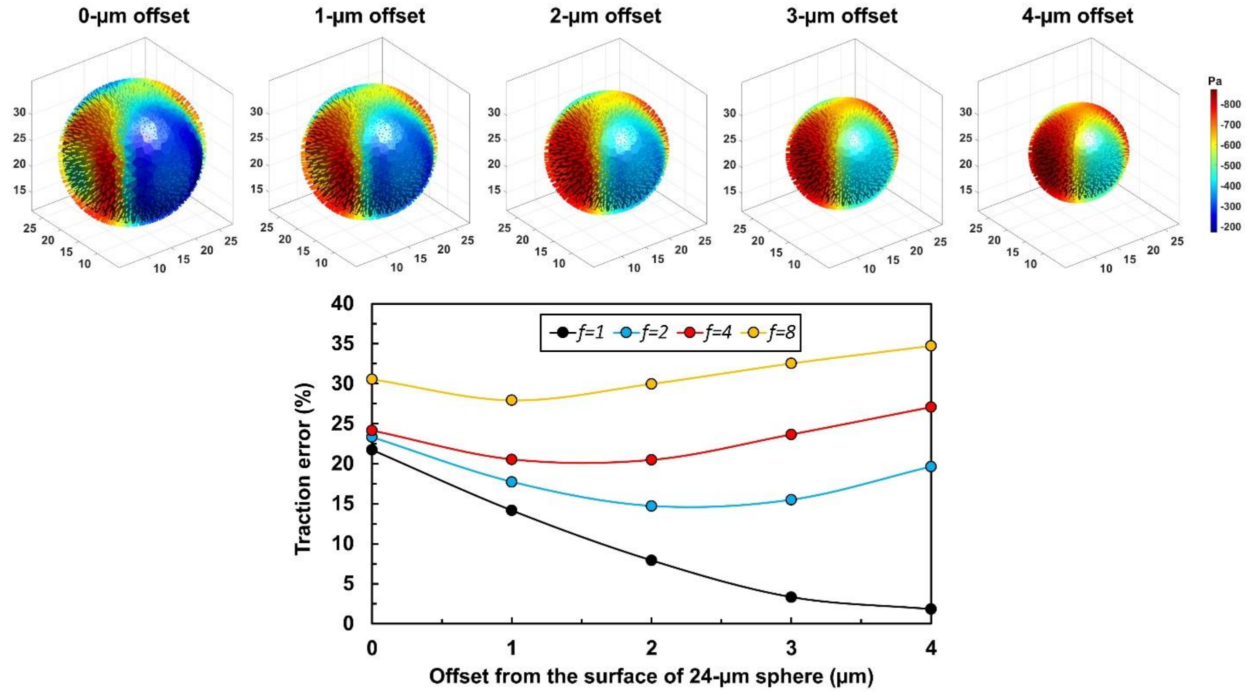

Supplementary Figure 20. Traction recovery sensitivity with respect to surface location of the microgel. Top, representative 3D recovered total traction calculated over the offset surfaces from the 24-μm sphere. **Bottom,** Plots of averaged percent traction error as a function of traction periodicity and surface location. Simulated 800/400 compression was applied on 24-μm sphere, and the traction was recovered and calculated on the surface of spheres with different offsets from the 24-μm sphere.

Supplementary References

1. Dobrynin, A. V., Colby, R. H. & Rubinstein, M. Scaling Theory of Polyelectrolyte Solutions. *Macromolecules* **28**, 1859-1871 (1995).

2. Ewoldt, R. H., Hosoi, A. & McKinley, G. H. New measures for characterizing nonlinear viscoelasticity in large amplitude oscillatory shear. *J. Rheology* **52**, 1427-1458 (2008).
